# Combined prophylactic and therapeutic immune responses against human papillomaviruses induced by a thioredoxin-based L2-E7 nanoparticle vaccine

**DOI:** 10.1371/journal.ppat.1008827

**Published:** 2020-09-04

**Authors:** Xueer Zhao, Fan Yang, Filipe Mariz, Wolfram Osen, Angelo Bolchi, Simone Ottonello, Martin Müller

**Affiliations:** 1 German Cancer Research Center, Heidelberg, Germany; 2 Department of Chemical Life Sciences and Environmental Sustainability, University of Parma, Parma, Italy; Fred Hutchinson Cancer Research Center, UNITED STATES

## Abstract

Global burden of cervical cancer, the most common cause of mortality caused by human papillomavirus (HPV), is expected to increase during the next decade, mainly because current alternatives for HPV vaccination and cervical cancer screening programs are costly to be established in low-and-middle income countries. Recently, we described the development of the broadly protective, thermostable vaccine antigen Trx-8mer-OVX313 based on the insertion of eight different minor capsid protein L2 neutralization epitopes into a thioredoxin scaffold from the hyperthermophilic archaeon *Pyrococcus furiosus* and conversion of the resulting antigen into a nanoparticle format (median radius ~9 nm) upon fusion with the heptamerizing OVX313 module. Here we evaluated whether the engineered thioredoxin scaffold, in addition to humoral immune responses, can induce CD8^+^ T-cell responses upon incorporation of MHC-I-restricted epitopes. By systematically examining the contribution of individual antigen modules, we demonstrated that B-cell and T-cell epitopes can be combined into a single antigen construct without compromising either immunogenicity. While CD8^+^ T-cell epitopes had no influence on B-cell responses, the L2 polytope (8mer) and OVX313-mediated heptamerization of the final antigen significantly increased CD8^+^ T-cell responses. In a proof-of-concept experiment, we found that vaccinated mice remained tumor-free even after two consecutive tumor challenges, while unvaccinated mice developed tumors. A cost-effective, broadly protective vaccine with both prophylactic and therapeutic properties represents a promising option to overcome the challenges associated with prevention and treatment of HPV-caused diseases.

## Author summary

Currently, there are three licensed prophylactic vaccines available against HPV, but none of them shows a therapeutic effect on pre-existing infections. Thus, a prophylactic vaccine also endowed with a therapeutic activity presents application potentials to individuals regardless of their HPV-infection status. Such a dual-purpose vaccine would be particularly valuable for post-exposure prophylaxis and shields population from recurrent HPV infections. Here, we constructed a combined vaccine relying on L2- and E7-specific epitopes grafted onto the surface of a hyper-stable thioredoxin scaffold. The resulting antigen was converted into a nanoparticle format with the use of a heptamerization domain. Our data document that the modular design of the antigen allows combination of B-cell and T-cell epitopes in one antigen without compromising either’s immunogenicity. The antigen retains its ability to provide broad protection against different HPV types but also presents strong therapeutic effects in a mouse tumor model. Therefore, the vaccine is potentially capable of resolving productive infection as well as HPV-related malignancies, and thus benefitting both uninfected and already infected individuals. Moreover, our vaccine utilizes *E*. *coli* as protein producer and distribution does not require cold-chain, which reduces costs making it applicable to less-affluent countries.

## Introduction

Cervical cancer is the fourth most common cancer in women worldwide. It is estimated that more than one million women are currently suffering from cervical cancer, and there were 570,000 new cases in 2018 [1]. According to current projections, the global burden of cervical cancer will continue to rise and will reach up to 700,000 cases and 400,000 deaths by 2030 [2, 3]. Nearly 90% of the current death cases occur in low-and middle income countries (LMIC) [2]. The main cause of cancerous cervical lesions is persistent infection by an oncogenic HPV type [4]. At least 14 oncogenic HPV types are known to induce cervical carcinogenesis [5]. While the carcinogenic process usually progresses from initial infection to the invasive carcinoma stage over one to three decades, precancerous lesions occur much earlier [6].

Currently, there are three licensed HPV prophylactic vaccines available, Gardasil4 (quadrivalent, HPV6/11/16/18), Cervarix (bivalent, HPV16/18), and Gardasil9 (nonavalent, HPV6/11/16/18/31/33/45/52/58). These vaccines are designed to induce protective, HPV type-specific antibodies to the major capsid protein L1 [7, 8]. However, despite their high prophylactic efficacy in HPV-naïve women, a therapeutic effect on pre-existing infections was not observed neither for Cervarix nor for Gardasil [9, 10]. Additionally, establishment of national HPV vaccination programs in the LMIC has been substantially constrained by the high cost and the complex supply-chain distribution of these heat-labile vaccines [11] (WHO, 2018).

An effective therapeutic strategy or post-exposure prophylaxis capable of eliminating HPV-infected cells should involve immune system activation with the aim to target viral antigens. An effective therapy should thus induce virus-specific T-cell responses. The HPV oncoprotein E7, due to its prominent role during the onset and progression of malignancy and expression limited to HPV-positive neoplastic cells, stands out among the most promising viral targets for vaccine-induced therapy [6, 12, 13]. Together with E6, the other major HPV oncoprotein, E7 has been the target of various immunotherapeutic approaches [13, 14]. Some of these have proven to be at least partially successful, but no immunotherapeutic protocol for the treatment of pre-cancerous lesions or established HPV tumors has thus far reached a clinically consolidated stage of development. A variety of E6/E7 antigen formulations has been set-up and tested, but only a few of them were actually aiming at both HPV prophylaxis and the immunotherapeutic treatment of pre-existing HPV-related malignancies [15–20]. Interestingly, some of these dual-purpose vaccine formulations, include HPV16 L2 as the monotypic prophylactic component of the vaccine, linearly fused to E7 alone [16–18, 21], or in combination with E6 [19, 20], in the form of either chimeric virus-like particles (VLPs) [16–18] or linear polypeptides [19, 20].

As cross-presentation represents a crucial step for the activation of T-cell responses, a therapeutic vaccine should rely on a highly immunogenic and possibly multivalent antigen for efficient delivery to dendritic cells. Following-up to our earlier work on the development of a broadly protective, multimeric L2-based vaccine prototype [22, 23] and to the encouraging results obtained with some of the above dual-purpose vaccines [15], we describe, here, the construction and testing of a combined vaccine relying on L2- and E7-specific epitopes grafted on the surface of a hyper-stable thioredoxin (Trx) scaffold. In particular, HPV-L2 immune reactivity was provided by a polytopic repeat derived from eight different HPV types [22, 23], coupled to the extended version of an E7-specific CTL epitope [24]. The resulting basic antigen was then converted into a nanoparticle format with the use of a heptamerization-promoting module (OVX313) that has previously proven to be a very effective super-scaffold for the construction of multivalent vaccines targeting various infectious agents in addition to HPV [25–29]. Our data document the pivotal role of the L2 8mer polytope and the OVX313 heptamerization domain in the induction of an E7-specific T-cell response, without any appreciable interference with the humoral production of HPV16 and HPV18 L2 neutralizing antibodies. We also provide a proof-of-concept demonstration of the strong therapeutic capacity of the Trx-8mer-flank E7-OVX313 vaccine in a double-challenge mouse model of HPV16 E7-induced carcinogenesis. Trx-8mer-flank E7-OVX313 thus lends itself as a robust and cost-effective vaccine with protective and therapeutic capacity, potentially capable of resolving productive infection as well as HPV-related malignancies, and thus benefitting both uninfected and already infected individuals.

## Results

### PfTrx promotes the induction of CD8^+^ cytotoxic T-cell responses against two unrelated T-cell epitopes

**Fig 1:**
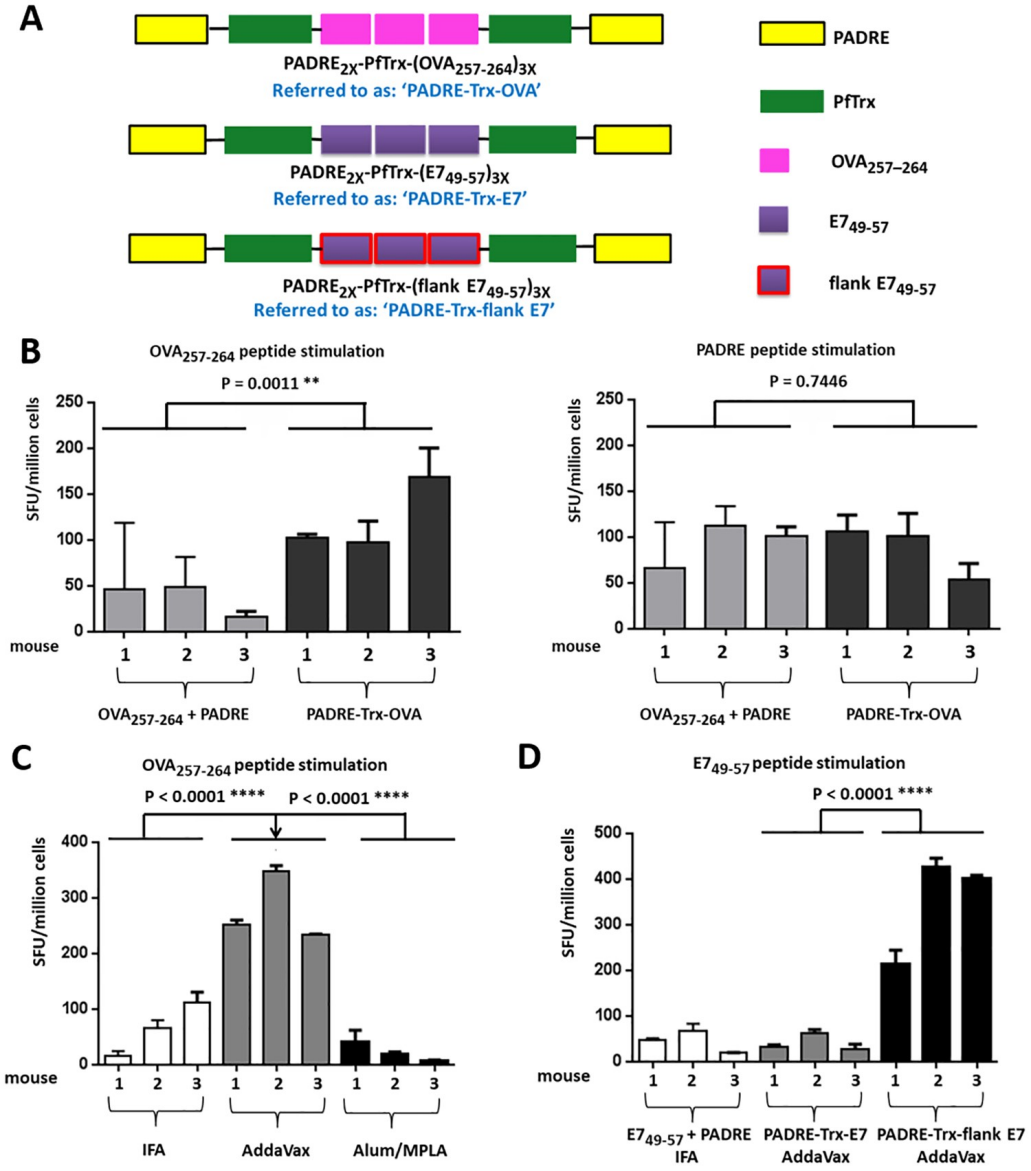
PfTrx-based vaccine constructs induce antigen-specific CD8^+^ T-cell responses. (A) Design of the constructs ‘PADRE-Trx-OVA’, ‘PADRE-Trx-E7’ and ‘PADRE-Trx-flank E7’. PADRE: universal CD4^+^ T-cell epitope, pan HLA DR-restricted in humans and I-A^b^-restricted in C57BL/6 mice [32]. PfTrx: thioredoxin scaffold from *Pyrococcus furiosus*. OVA_257–264_: ovalbumin-derived CTL epitope (SIINFEKL, H2-K^b^ restricted). HPV16 E7_49–57_: HPV16 E7-derived CTL epitope (RAHYNIVTF, H2-D^b^ restricted) [24]. Flank E7_49-57_: extended E7_49-57_ epitope, flanked on both sides by the five amino acids that in the HPV16 E7 protein are located upstream (QAEPD) and downstream (CCKCD) to the sequence of the E7_49-57_ epitope (RAHYNIVTF). (B) Numbers of IFN-γ spots per 10^6^ splenocytes (shown as SFU (spot-forming unit)/million cells) are compared among groups of mice immunized with OVA_257-264_ and PADRE peptide mix or the PADRE-Trx-OVA protein, respectively. Both groups were adjuvanted with IFA (Incomplete Freund’s adjuvant). Splenocytes were stimulated *in vitro* with either the OVA_257-264_ or the PADRE peptide. (C) Comparison of different adjuvants. Numbers of IFN-γ spots per 10^6^ splenocytes are compared in three groups of mice vaccinated with PADRE-Trx-OVA formulated with different adjuvants (IFA, AddaVax or Alum/MPLA). Splenocytes were stimulated with the OVA_257-264_ peptide. (D) Influence of amino acids flanking the E7_49-57_ epitope. Mice were immunized with HPV16 E7_49-57_ and PADRE peptides mix formulated with IFA, or PADRE-Trx-E7 or PADRE-Trx-flank E7 formulated with AddaVax. Splenocytes were stimulated with the HPV16 E7_49-57_ peptide. Shown are the mean and SD (standard deviation) of triplicate values on each mouse. P-value ≤ 0.05 was considered as significant and are labeled as follows: *, P-value < 0.05; **, P-value < 0.01; ***, P-value < 0.001; ****, P-value < 0.0001.

The PfTrx scaffold, a modified derivative of the thioredoxin protein from the hyperthermophilic archaeon *Pyrococcus furiosus* [40], is well suited for insertion of L2 polytopes resulting in a potent induction of neutralizing antibody responses that provide broad protection against multiple, and practically all oncogenic, HPV types [23, 30, 31]. To determine whether PfTrx also leads to the induction of cell-mediated immune responses, we inserted into the display site of thioredoxin three copies of the strong H2-K^b^-restricted CD8^+^ T-cell (CTL) epitope OVA_257-264_ or three copies of the H2-D^b^-restricted epitope HPV16 E7_49-56_ [24] (Fig 1A). We added the universal T-helper epitope PADRE [32] to both ends of the thioredoxin moiety of the antigens to compensate a possible lack of I-A^b^-restricted CD4^+^ T-cell epitopes (Fig 1A). As a positive control, mice were immunized with a mixture of the PADRE peptide and either the OVA- or the E7- CTL epitope peptides formulated in incomplete Freund’s adjuvant (IFA). Seven days after immunization, splenocytes were analyzed by IFN-γ ELISpot assays using the OVA, E7, or the PADRE peptides as stimulants. As shown in Fig 1B, PADRE-Trx-OVA immunization led to stronger responses against the OVA-specific CTL epitope compared to control peptide immunization (Fig 1B, left), while a similar response to protein and peptide immunization was observed in the case of stimulation with the PADRE peptide (Fig 1B right). This indicates that the PADRE-containing PfTrx scaffold is an efficient inducer of specific CTL-responses. We also tested different immune-adjuvants as formulation supplements of the PADRE-Trx-OVA antigen. As shown in Fig 1C, the MF59-like, squalene-based adjuvant AddaVax elicited very robust responses against the OVA-specific CTL epitope, and outperformed both the IFA and the Alum/MPLA adjuvants. IFA, instead, proved to be a superior adjuvant for peptide immunization (S1 Fig). AddaVax was thus used as adjuvant in all subsequent experiments performed with PfTrx-based antigens. As shown in Fig 1D, even with AddaVax as adjuvant, immunogenicity of the E7 epitope bearing Trx construct (PADRE-Trx-E7; see Fig 1A), was not significantly higher than that of the corresponding free peptides. In this construct, the three-fold repeated E7 epitopes are separated by a Gly-Gly-Pro linker and we reasoned that there might be insufficient proteasomal epitope processing. Therefore, a second antigen (PADRE-Trx-flank E7) was designed, in which the E7 epitopes were individually flanked, both C- and N-terminally, by the five amino acids that in the HPV16 E7 protein are located upstream and downstream to the sequence of the standard E7_49-57_ epitope (see Fig 1A legend for details). As shown in Fig 1D, a robust response, outperforming the peptide-induced responses, was observed against the HPV16 E7-specific CTL epitope upon immunization with the PADRE-Trx-flank E7 antigen. In conclusion, the PADRE-supplemented PfTrx scaffold appears to be an effective inducer of cytotoxic T-cell responses and was thus considered as a promising building block for the construction of a therapeutic vaccine.

### L2 neutralization epitopes can be combined with CTL epitopes

**Fig 2:**
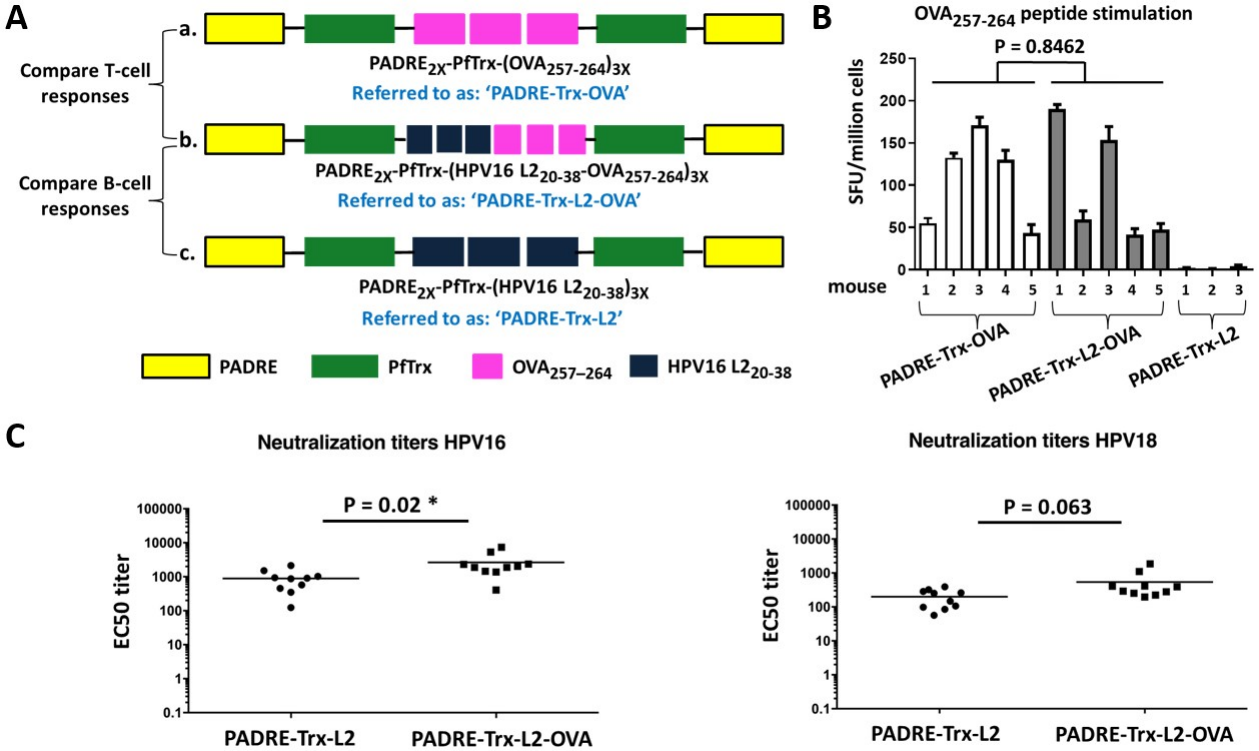
OVA-specific T-cell epitopes and HPV16 L2-specific B-cell epitopes can be combined in a single construct without compromising each other’s immunogenicity. (A) Antigen structure. Design a: Construct PADRE-Trx-OVA only containing the T-cell epitope OVA_257-264_. Design b: Construct PADRE-Trx-L2-OVA bearing both the T-cell epitope OVA_257-264_ and the B-cell epitope HPV16 L2_20-38_. Design c: Construct PADRE-Trx-L2 only containing the HPV16 L2_20-38_ B-cell epitope. (B) Influence of L2 B-cell epitopes on the induction of OVA-specific CTL responses. Numbers of IFN-γ spots per 10^6^ splenocytes are compared in three groups of mice immunized with PADRE-Trx-OVA, PADRE-Trx-L2-OVA or PADRE-Trx-L2 (negative control). Splenocytes were stimulated with the OVA_257-264_ peptide. Shown are the mean and SD of triplicate values on each mouse. (C) Influence of OVA epitopes on the induction of anti-HPV16 and anti-HPV18 neutralizing antibodies. Sera were collected one month after the last immunization with PADRE-Trx-L2 or PADRE-Trx-L2-OVA (10 mice/group) and analyzed against HPV16 and HPV18 pseudovirions using the L1-PBNA. Each symbol represents the neutralization titer (EC50) of an individual animal; geometric means of the titers for each group are indicated by horizontal lines. P-value ≤ 0.05 was considered as significant (*).

After assessing the potential of PfTrx as a scaffold capable of inducing CD8^+^ cytotoxic T-cell responses, we asked whether L2 neutralization epitopes could be incorporated in the construct in order to induce a B-cell response without compromising the ability to drive T-cell immunity. The L2 proteins of papillomaviruses harbor a major cross-neutralization epitope in their N-terminal region, corresponding to amino acids 20–38 of HPV16 L2 [33]. Starting with the PADRE-Trx-OVA construct, we added three copies of the HPV16 L2_20-38_ epitope (Fig 2A) and used this combined antigen to immunize C57BL/6 and BALB/c mice.

To confirm the ability to induce CTL responses, we performed IFN-γ ELISpot assays after a single immunization of C57BL/6 mice with PADRE-Trx-OVA and PADRE-Trx-L2-OVA constructs, using PADRE-Trx-L2 as a negative control. As shown in Fig 2B, where the cellular immunity induction performance of PADRE-Trx-L2-OVA is compared with that of the L2-lacking construct PADRE-Trx-OVA, insertion of the L2 epitopes did not significantly affect the ability to induce OVA-specific CTL responses. The ability of the combined vaccine to induce a L2-targeted, anti-HPV humoral response was determined, in parallel, in PADRE-Trx-L2 and PADRE-Trx-L2-OVA immunized BALB/c mice. As shown by the results of the anti-HPV16 and HPV18 pseudovirion-based neutralization assays in Fig 2C, the presence of the OVA-derived CTL epitopes slightly enhanced the induction of neutralizing antibodies, although this effect was only statistically significant in the case of HPV16 pseudovirions.

### Antigen heptamerization and addition of an L2 polytope within the display site of thioredoxin boost T- and B-cell immunogenicity

**Fig 3:**
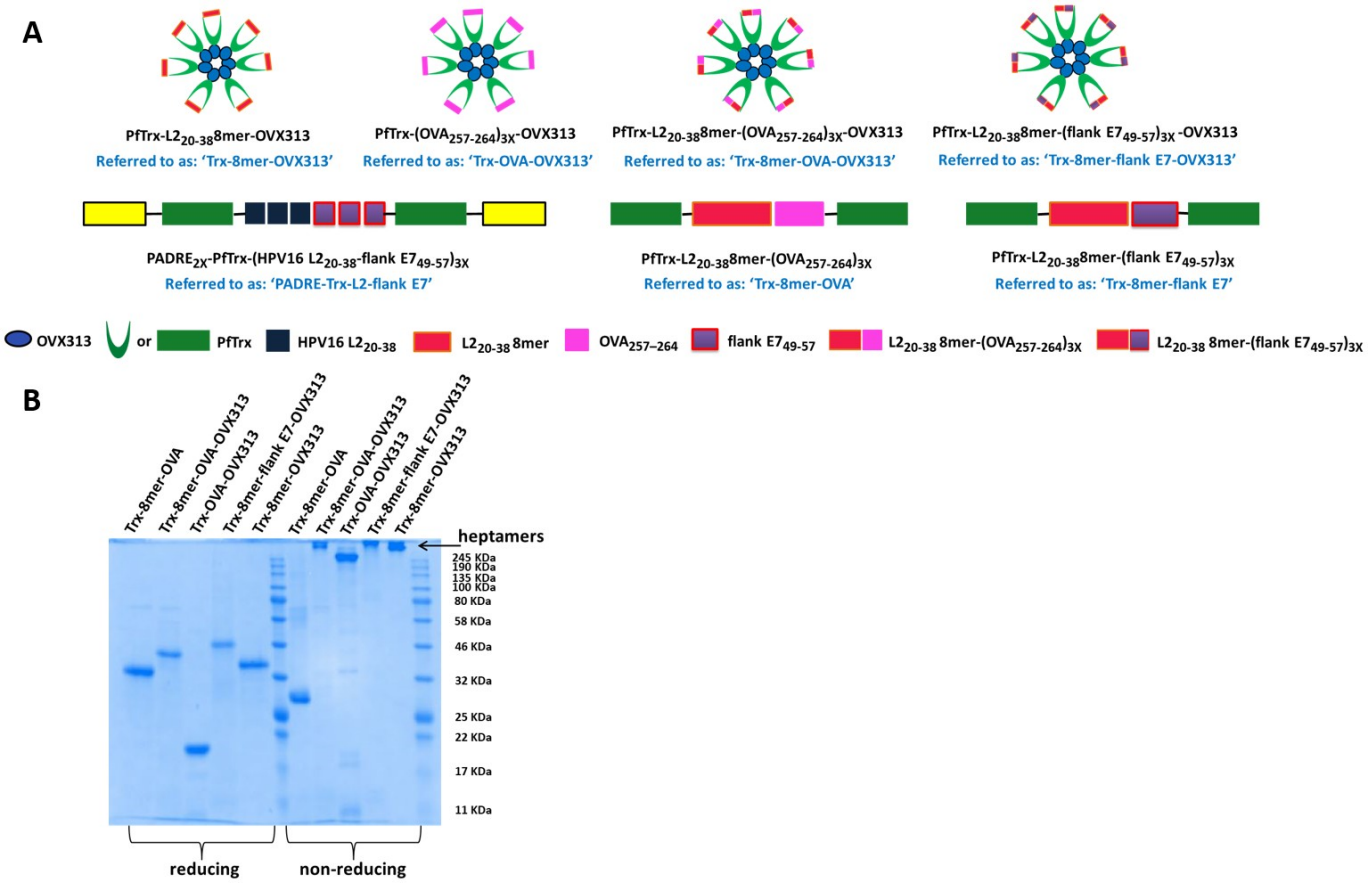
Addition of the OVX313 domain leads to multimerization of the antigens. (A) Antigenic constructs used for immunization. OVX313: Chimeric version of the avian C4-binding protein heptamerization domain (OligoDOM technology, OSIVAX). L2_20-38_ 8mer: polytope comprising the L2_20-38_ epitopes from eight different HPV types (16-18-31-33-35-6-51-59), selected on the basis of sequence homology to the major cross-neutralization HPV16 L2_20-38_ epitope. (B) SDS-PAGE analysis of the purified antigens under reducing (left) and non-reducing conditions (right). Note: the molecular weight shift of the monomer Trx-8mer-OVA results from intensive L2 epitope-related formation of disulfide bonds in the non-reducing conditions.

**Fig 4:**
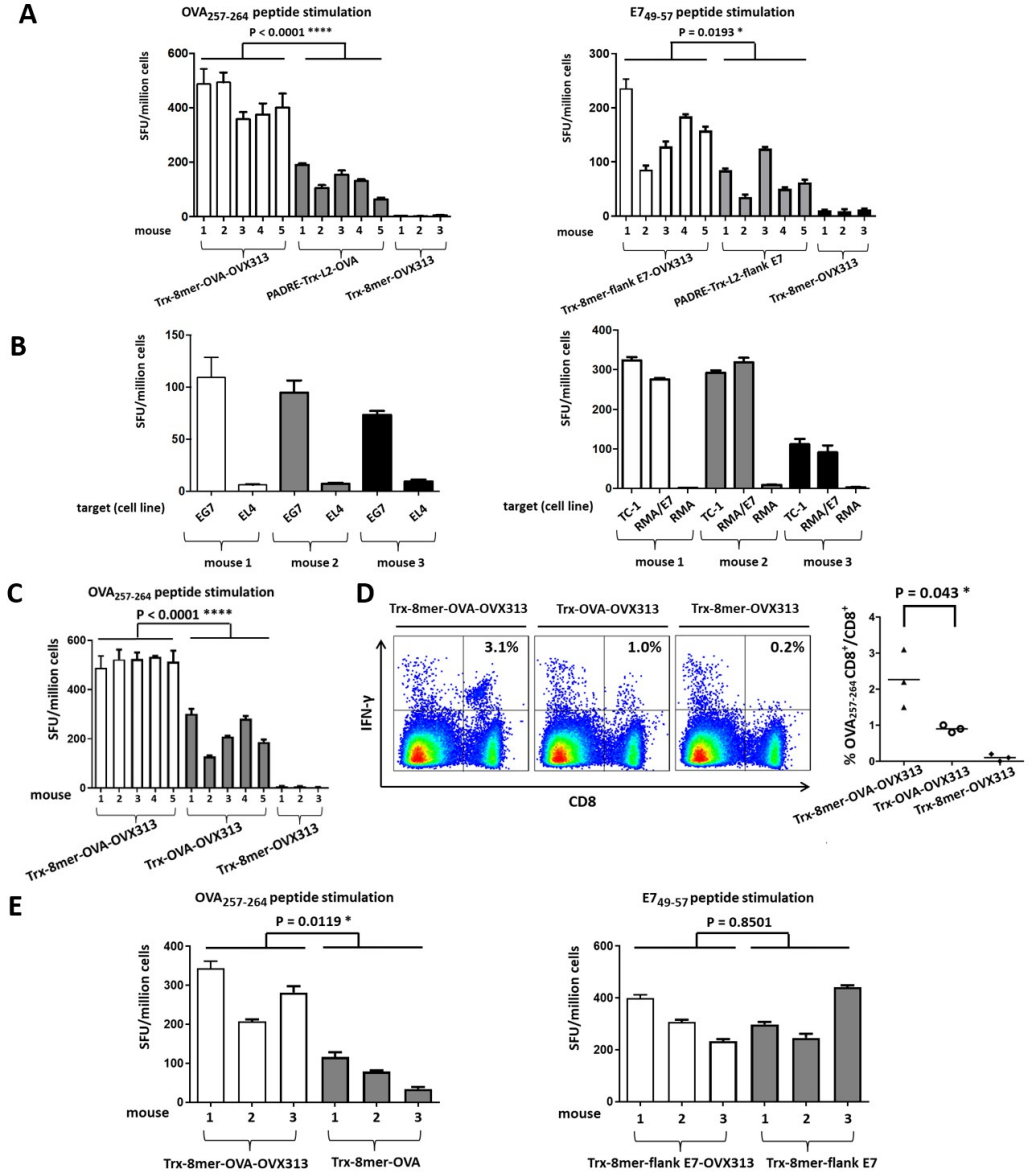
Heptamerization and addition of the L2 polytope boosts T-cell immunogenicity. (A) IFN-γ ELISpot analysis of the T-cell responses induced by the heptameric Trx-8mer-OVA-OVX313 (left) and Trx-8mer-flank E7-OVX313 (right) antigens compared to the responses elicited by the corresponding monomeric PADRE-Trx-L2-OVA and PADRE-Trx-L2-flank E7 antigens; the Trx-8mer-OVX313 construct served as a negative control. Splenocytes were stimulated with either the OVA_257-264_ or the E7_49-57_ peptide, as indicated. (B) Recognition of OVA-expressing EG7 cells (left) or E7-expressing RMA/E7 transfectants and TC-1 cells (right) by splenocytes from C57BL/6 mice immunized with the Trx-8mer-OVA-OVX313 (left) or the Trx-8mer-flank E7-OVX313 (right). Parental EL4 cells (left) and RMA cells (right) served as negative controls; the bar colors refer to the different animals utilized as 'biological replicates' in these experiments, as indicated. (C) Presence of the 8mer polytope induces stronger OVA-specific T-cell responses. During the assay, splenocytes were stimulated with the OVA_257-264_ peptide. Shown are the mean and SD of triplicate values on each mouse. (D) Absolute number of OVA-specific CD8^+^ T cells among splenocytes from immunized mice determined by flow cytometry using H2-K^b^/OVA_257-264_ (SIINFEKL) tetramers. Representative examples from three different mice are shown on the left; the percentages of IFN-γ-producing CD8^+^ T cells are reported in the upper right quadrant. The diagram on the right shows the cumulative data obtained from the experiments performed with the tetramers; each symbol represents one mouse; the mean percentage (OVA_257-264_–specific CD8^+^ T cells/total CD8^+^ T cells) is indicated by horizontal bars. (E) Influence of the OVX313 domain on OVA- and E7-specific CTL responses. In the left graph, the numbers of IFN-γ spots per 10^6^ splenocytes, measured upon stimulation with the OVA_257-264_ peptide, are compared in two groups of mice immunized with Trx-8mer-OVA-OVX313 or Trx-8mer-OVA, as indicated. The results of the same assay, performed with splenocytes from Trx-8mer-flank E7-OVX313 or Trx-8mer-flank E7 immunized mice, stimulated with the E7_49-57_ peptide, are shown on the right. Shown are the mean and SD of triplicate values on each mouse. P-value ≤ 0.05 was considered as significant and are labeled as follows:*, P-value < 0.05; **, P-value < 0.01; ***, P-value < 0.001; ****, P-value < 0.0001.

According to our previous results [30, 33], incorporation into PfTrx of only the major L2 cross-neutralization epitope of HPV16 fails to provide protection against certain oncogenic HPV types, some of which, for example HPV31 and 51, partially escape neutralization by the antibodies elicited by HPV16 L2 [30, 33]. Therefore, in order to induce a more comprehensive humoral response against all oncogenic mucosal HPV types plus the mucosal low-risk types HPV6 and 11 which are responsible for > 80% of genital warts, we developed a polytope comprising the L2 epitopes of seven oncogenic HPV types plus HPV6 (named 8mer [22, 23]). When coupled with the heptamerization domain OVX313, the resulting antigen (Trx-8mer-OVX313) exhibited a broader and stronger cross-protection in mice and guinea pigs compared to its monomeric version (Trx-8mer) [23]. Additionally, we documented the presence of a T-helper epitope derived from the OVX313 domain in BALB/c mice [23]. Based on this body of evidence, we set out to replace the promiscuous T-helper epitope PADRE and the HPV16 L2 trimer with the OVX313 module and the 8mer polytope, respectively (see Fig 3), in order to determine whether the resulting construct would lead to an increased T-cell response. As shown in Fig 4A, compared to the monomeric PADRE-Trx-L2-OVA antigen, the heptameric form of the antigen containing the 8mer polytope (Trx-8mer-OVA-OXV313) induced a stronger OVA-specific T-cell response. In the IFN-γ ELISpot assay, the stimulatory capacity of the double-modified Trx-8mer-OVA-OXV313 antigen was increased by more than two-fold. Incorporation of the OVX313 domain together with the 8mer polytope into the antigen harboring the E7 multiepitope (Trx-8mer-flank E7-OVX313, Fig 3) also positively affected T-cell induction, albeit to a lesser extent compared to the OVA antigen (Fig 4A). Importantly, as shown in Fig 4B, both heptameric constructs induced T-cell responses specifically recognizing syngeneic target cells endogenously expressing OVA or E7, including TC-1 cells, thereby demonstrating the capacity of these vaccines to prime tumor-reactive T cells *in vivo*.

Next, we asked whether the presence of the L2 polytope, the OVX313 domain, or both, also leads to an enhanced induction of CD8^+^ T-cell responses. To directly document a possible contribution of the L2 polytope, we compared the immunogenicity of the Trx-8mer-OVA-OVX313 and the Trx-OVA-OVX313 constructs (see Fig 3) in C57BL/6 mice. According to the results of the IFN-γ ELISpot assay in Fig 4C and the OVA-specific tetramer staining data shown in Fig 4D, the presence of the L2 polytope in the heptamerized antigen substantially increased the immunogenicity of the OVA-derived epitopes. In both assays, a more than two-fold increase in spot numbers and in the frequency of OVA-specific CD8^+^ T cells was observed with splenocytes from mice immunized with the antigen harboring the 8mer polytope. To better delineate the importance of multimerization on the increased immunogenicity induced by the OVX313 module, in a complementary approach, we further compared the immune performance of the Trx-8mer-OVA-OVX313 and the Trx-8mer-OVA antigens (see Fig 3 for details on construct design). As shown in Fig 4E, the presence of the OVX313 module significantly improved OVA-specific CTL responses. In contrast, no such effect was observed for constructs bearing the E7 epitopes, for which the induction of an E7-specific CTL response did not differ significantly between the Trx-8mer-flank E7-OVX313 and the Trx-8mer-flank E7 antigens.

**Fig 5:**
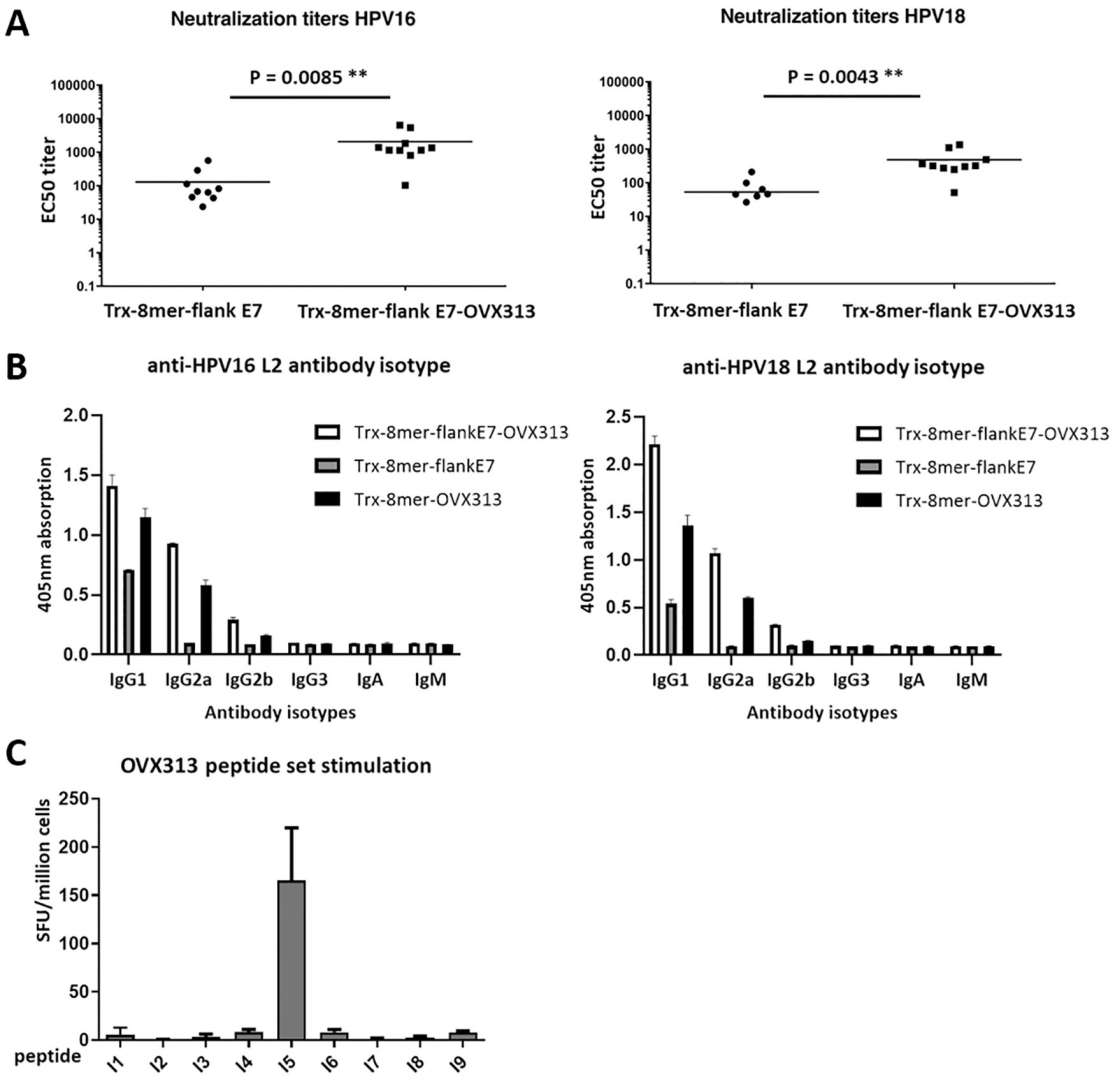
OVX313 domain enhanced generation of L2-specific antibodies. (A) Sera collected from vaccinated mice (10 animals per group) one month after the last immunization with Trx-8mer-flank E7 or Trx-8mer-flank E7-OVX313, were analyzed against HPV16 (left) and HPV18 (right) pseudovirions using the L1-PBNA. Each symbol represents the neutralization titer (EC50) of an individual mouse; geometric means of the titers for each group are indicated by horizontal lines. EC50 values of three Trx-8mer-flank E7 immunized mice are lower than 50 (the EC50 value set as threshold for neutralization positivity); the corresponding data are thus not shown in the graph. P-value ≤ 0.05 was considered as significant. *, P-value < 0.05; **, P-value < 0.01. (B) Analysis of isotype distribution of L2-specific antibodies measured by antibody isotype ELISA; the values are the mean ±SD of data obtained from duplicate assays performed on the pooled sera from each immunization group. (C) A T-helper response is induced by the ‘OVX313-I5’ peptide. Mice were immunized twice at 5 days intervals with the Trx-8mer-flank E7-OVX313 antigen. The splenocytes were stimulated with the OVX313 peptide panel (OVX313-I1 to OVX313-I9; see ‘Materials and methods‘ for details). Data, representing the numbers of IFN-γ spots per 10^6^ splenocytes, are the mean ±SD of three mice for each peptide stimulation.

Nevertheless, in keeping with the results of our previous studies, we observed a clear contribution of OVX313-induced heptamerization to the strength of the L2-specific antibody responses. Indeed, as shown in Fig 5A, neutralizing antibody titers against HPV types 16 and 18 were approximately 10-15-fold higher in mice immunized with the heptameric antigen (Trx-8mer-flank E7-OVX313) compared to its monomeric counterpart (Trx-8mer-flank E7). The two antigens also differed in the isotype profile of the elicited antibodies. In fact, as shown in Fig 5B, a distribution with a prevalent IgG1 isotype, that correlates with a Th2 response, was observed for the OVX313-lacking antigen, whereas a pattern skewed toward the Th1 response–correlated IgG2a and IgG2b isotypes was apparent for the OVX313-containing antigens, regardless of the presence of the E7 epitope. A similar isotype distribution of L2-specific antibodies was observed for both HPV16 (Fig 5B, left panel) and HPV18 (Fig 5B, right panel). Based on this data, we conclude that OVX313-mediated heptamerization enhances both humoral and cellular immunogenicity, even though the latter effect is influenced by the specific CTL epitope.

We then wished to find out whether the enhanced immunogenicity observed in the presence of the heptamerization module only depends on OVX313-induced antigen multimerization or also involves an improved T-helper cell activation. To this end, we used a set of long overlapping peptides spanning the entire OVX313 sequence and, in keeping with our previous data [23], we identified a putative T-helper epitope located between amino acids 8 and 27 (VCGEVAYIQSVVSDCHVPTA; OVX313-I5) (Fig 5C). The OVX313 module thus may contribute to the observed enhancement of immunogenicity also by improving T-helper activation.

**Fig 6:**
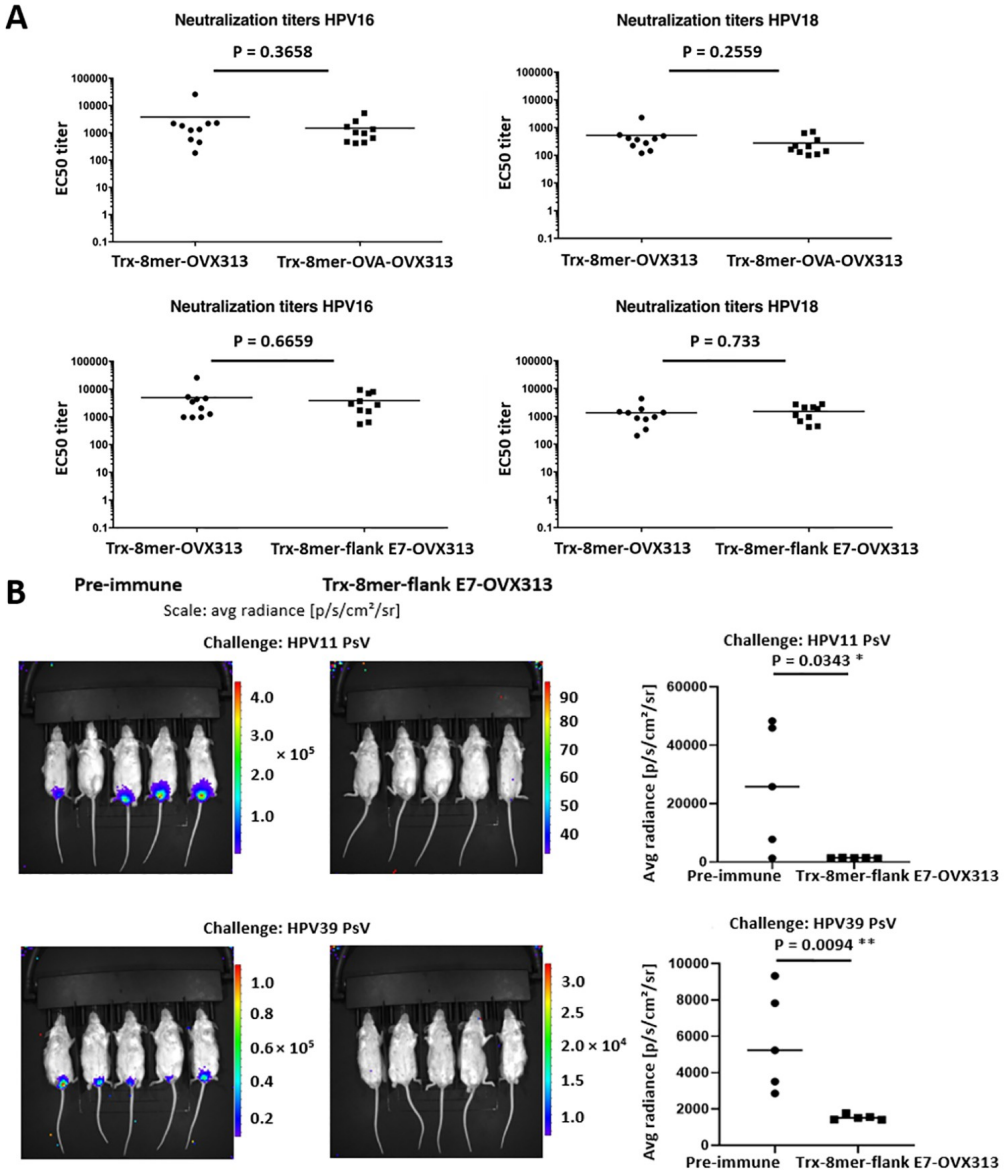
The antigen Trx-8mer-flank E7-OVX313 induces robust humoral immune responses. (A) The presence of the OVA- or flank E7-specific epitopes does not compromise the induction of L2-specific B-cell responses. Sera collected from mice (10 animals/ group) one month after the last immunization with Trx-8mer-OVX313, Trx-8mer-OVA-OVX313 or Trx-8mer-flank E7-OVX313, were analyzed against HPV16 and HPV18 pseudovirions using the L1-PBNA. Each symbol represents the neutralization titer (EC50) of an individual mouse; geometric means of the titers for each group are indicated by the horizontal lines. (B) *In vivo* protection afforded by anti-Trx-8mer-flank E7-OVX313 sera. Sera from nonimmunized animals were used as control. Sera from 10 mice immunized with the Trx-8mer-flank E7-OVX313 antigen were pooled and passively transferred into naive mice. Recipient animals were then challenged with HPV11 and HPV39 PsVs. Images of *in vivo* challenge show the magnitude of vaginal infection by HPV11 and 39 PsVs. The color scales indicate the intensity of luciferase expression; a region-of-interest (ROI) analysis for average radiance (photons per second per square centimeter per steradian) was performed using the Living Image 2.50.1 software. Note that due to the different *in vivo* transduction activities of the various HPV PsV types, different scales were used. The diagrams on the right show the analytical data obtained from the experiments. Each symbol represents the average radiance of an individual mouse with the horizontal lines indicating the geometric means. P-value ≤ 0.05 was considered as significant. *, P-value < 0.05; **, P-value < 0.01.

The above data, together with the lack of interference of the CTL epitopes with the humoral responses induced by the 8mer component in a single construct shown by PBNA (Fig 6A) and also by protection in the cervico-vaginal mouse model (Fig 6B), clearly point to the potential of the Trx-8mer-CTL epitope-OVX313 design for constituting a prophylactic/therapeutic-combined anti-HPV vaccine.

### Trx-8mer-flank E7-OVX313 antigen promotes tumor regression in a mouse model of HPV16-induced carcinogenesis

**Fig 7:**
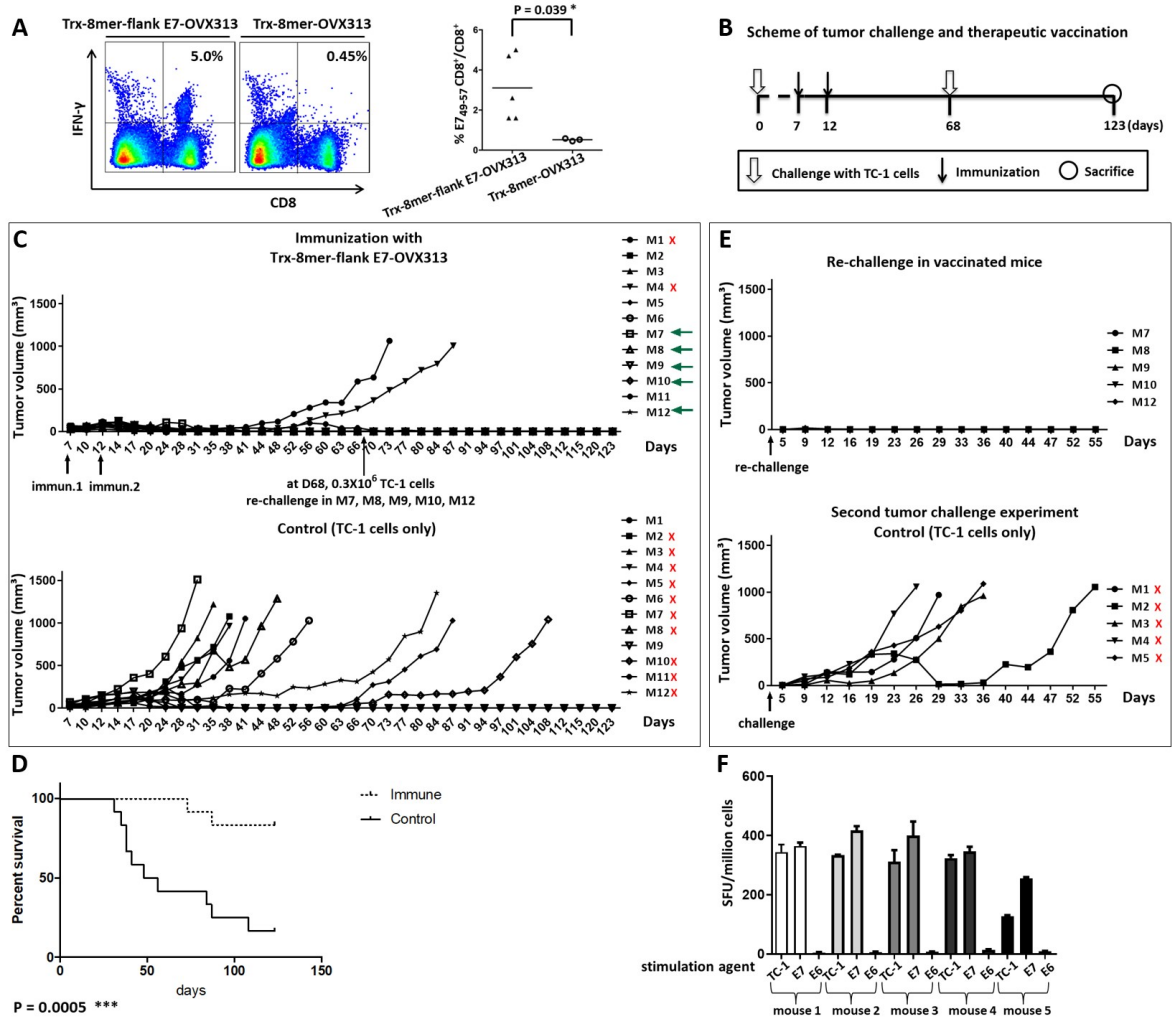
Vaccination with Trx-8mer-flank E7-OVX313 suppresses the outgrowth of grafted TC-1 tumors in mice. (A) E7-specific CD8^+^ T-cell responses were evaluated using H2-D^b^/E7_49-57_ streptamer staining. Splenocytes from immunized mice were double-stained with an anti-CD8-PE antibody and an APC-conjugated H2-D^b^/E7_49-57_ streptamer and visualized by flow cytometry. Representative raw data for two mice are shown in the left panels; the percentages of IFN-γ-producing CD8^+^ T cells are shown in the upper right quadrant. The diagram on the right shows the cumulative data obtained from the experiments performed with the streptamer; each symbol represents one mouse; the mean percentage (E7_49-57_–specific CD8^+^ T cells/total CD8^+^ T cells) is indicated by horizontal bars. (B) Experimental scheme of tumor challenge and therapeutic vaccination. (C) Tumor growth curves of vaccinated and unvaccinated mice (12 mice per group). (D) Animal survival rate shown by Kaplan-Meier curves; the Log-rank test indicates a significant difference in survival (p = 0.0005). (E) Tumor growth kinetics after re-challenge. Sixty-eight days after inoculation of tumor cells, five vaccinated mice (indicated by green arrows in Fig 7C) were re-challenged with 0.3×10^6^ TC-1 cells. As a reference and internal control, five naïve mice were implanted in parallel with the same amount of TC-1 cells. Red crosses indicate mice that had to be sacrificed ahead of time due to an excessive tumor burden (tumor diameter over 1.5 cm). (F) A potent E7-specific T-cell response is apparent in vaccinated, tumor-regressed mice. Numbers of IFN-γ spots per 10^6^ splenocytes are determined. Splenocytes were stimulated with TC-1 cells, E7_49-57_ or E6_48-57_ [37] peptide. Shown are the mean and SD of triplicate values on each mouse under the specific stimulation agent.

We initially determined the magnitude of the E7-specific CTL responses induced by two immunizations of C57BL/6 mice with the AddaVax-adjuvanted heptameric construct. As shown in Fig 7A, using fluorochrome-conjugated streptamers, up to 5% of the CD8^+^ T-cell population (with an average value of 3.2%; right panel) was found to be specific for the E7 epitope, thus indicating a potent E7-specific immunogenicity of the Trx-8mer-flank E7-OVX313 antigen.

**Fig 8:**
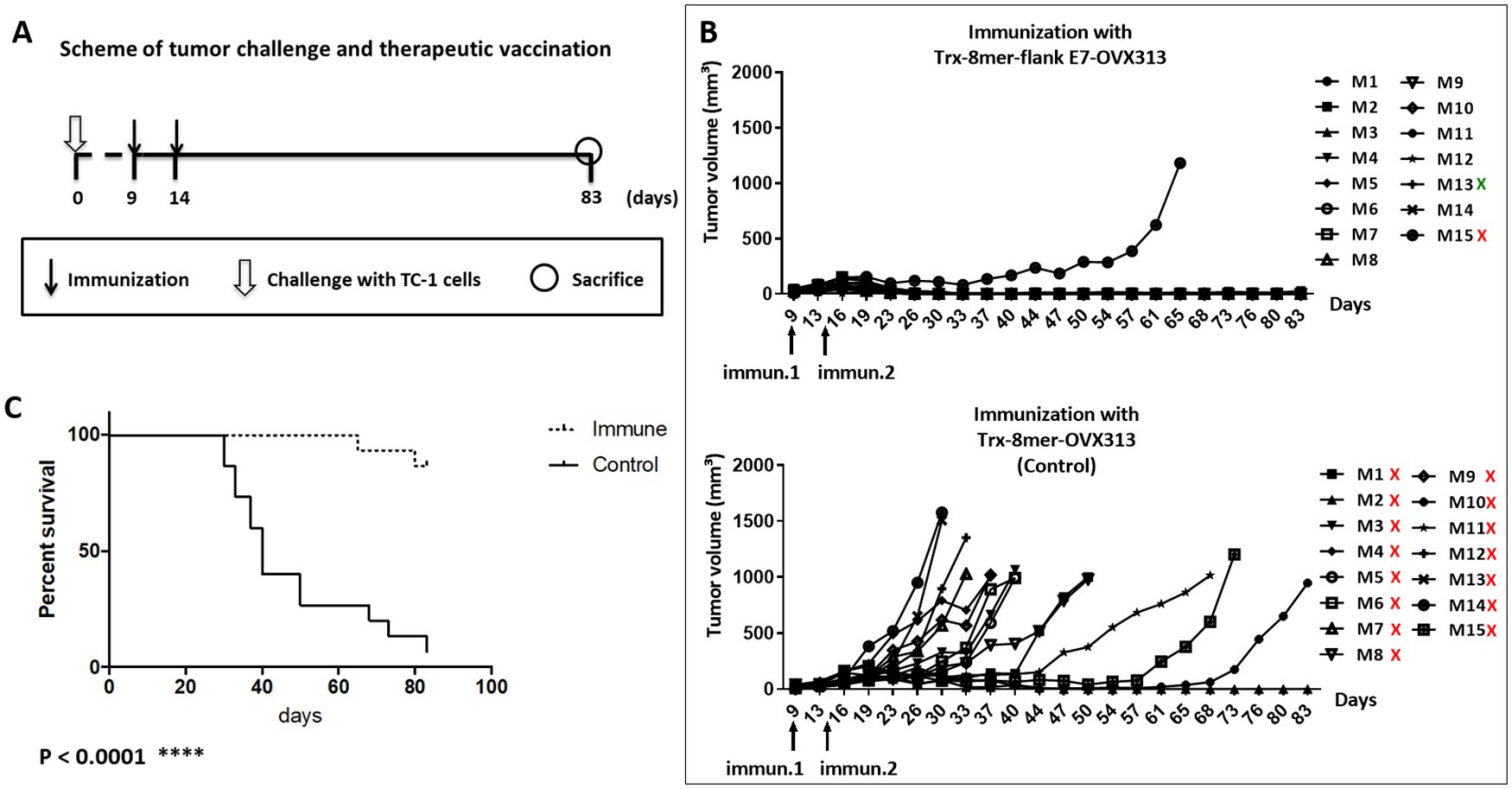
Vaccination with Trx-8mer-flank E7-OVX313 induces regression of advanced TC-1 tumors. (A) Experimental scheme of tumor challenge and therapeutic vaccination. Specifically, mice were inoculated with 0.3×10^6^ TC-1 cells suspended in 100 μl of PBS on their right flank. When tumor size reached a diameter of 4-6mm, half of the tumor-bearing animals were immunized subcutaneously at the base of the tail with 20 μg of the Trx-8mer-flank E7-OVX313 antigen, whereas the remaining tumor-bearing mice received 20 μg of the control Trx-8mer-OVX313 antigen. Both antigens were adjuvanted with AddaVax, and two vaccine doses were administered 9 and 14 days after the challenge. Mice were excluded from the experiment when tumor diameter exceeded 1.5 cm. (B) Tumor growth curves of Trx-8mer-flank E7-OVX313 and Trx-8mer-OVX313 immunized mice (15 mice per group). (C) Animal survival rate displayed as Kaplan-Meier curves; the Log-rank test indicates a significant difference in survival (p<0.0001). Red crosses indicate mice that had to be sacrificed ahead of time due to an excessive tumor burden (tumor diameter over 1.5 cm). The mouse indicated by the green cross was sacrificed owing to skin lesions irrelevant to the tumor.

Having demonstrated the CTL immunogenicity of our combined antigen *ex vivo*, we next tested its therapeutic capacity in the TC-1 murine model of HPV16 E7-induced carcinogenesis. To this end, C57BL/6 mice were first challenged subcutaneously with E7-expressing syngeneic TC-1 cells [34], followed by two immunizations with the Trx-8mer-flank E7-OVX313 antigen 7 and 12 days after the challenge (see Fig 7B for a schematic representation of this experiment). As indicated by the tumor growth and Kaplan-Meier survival curves reported in Fig 7C and 7D, 10 out of 12 vaccinated mice remained tumor-free, whereas 10 out of 12 control mice developed tumors within about two months. Moreover, complete protection was observed in five mice that had successfully responded to Trx-8mer-flank E7-OVX313 immunotherapy and were re-challenged with TC-1 cells. In contrast, all mice of the same age belonging to the non-vaccinated control group developed tumors (Fig 7E). These results clearly indicate that the T-cell responses we have found to be induced by the Trx-8mer-flank E7-OVX313 antigen (see Fig 7A) are sufficient not only to induce regression of TC-1 tumors, but also to prevent tumor outgrowth upon subsequent re-challenge. We then tested the CTL responses of cured mice that had remained tumor free upon re-challenge by *ex-vivo* IFN-γ ELISpot assays and included an E6-derived CTL epitope in addition to the E7-specific epitope in order to test for possible epitope spreading [35, 36]. A potent E7-specific T-cell response, but no response against E6, was detected in these animals (Fig 7F). To further evaluate therapeutic efficacy, tumor-bearing mice at a further advanced stage were immunized with either the Trx-8mer-flank E7-OVX313 antigen, or with the E7-lacking, Trx-8mer-OVX313 construct as a control, (Fig 8A). As reported in Fig 8B and 8C, a curbed tumor growth and an improved survival rate were observed in mice immunized with the Trx-8mer-flank E7-OVX313 antigen compared to the Trx-8mer-OVX313 control. In conclusion, vaccination with Trx-8mer-flank E7-OVX313 exerted a strong therapeutic effect in the TC-1 tumor model of HPV16-induced carcinogenesis.

## Discussion

Not many diseases highlight how global inequity impacts humanity as cervical cancer. Currently available measures for prevention, including prophylactic vaccination, have being poorly implemented on a national scale in low and middle income countries, which resulted in twice and three times higher cervical cancer incidence and mortality, respectively, compared to high-income countries, where such measures are widely available. In addition, a vast number of already infected women are expected to develop cervical precancerous and cancerous lesions in the years to come. While there is no approved immunotherapy for HPV-driven malignancies, current treatments vary from surgical intervention, chemotherapy, radiation, to a combination of these three approaches, which although often effective, do not clear HPV infection. In this context, a prophylactic vaccine also endowed with a therapeutic activity, and thus administrable to exposed individuals (e.g., HIV-positive subjects) regardless of their HPV-infection status would be highly desirable. Such a dual-purpose vaccine would be particularly valuable for post-exposure prophylaxis. Early stages of HPV infection are characterized by the predominant expression of virus early proteins (including E6 and E7), which do not sustain virion production, and very little or no expression of late capsid proteins (including L2). Under these conditions, anti-early HPV protein, in particular anti-E6 or anti-E7 T-cell responses are critical for clearing virus-infected cells, whereas humoral antibody responses to capsid proteins have no proven effect against a prevalent infection. This scenario changes within days as virus-infected cells follow-on in the epithelial differentiation program, with the overexpression of virus capsid proteins and subsequent virion formation in the upper layers of the epithelium (upon skin desquamation). Infection of other epithelial cells by these newly released virions can only be prevented by humoral antibody responses directed against capsid proteins, while cellular immunity does not play a significant role at this stage of the viral replication cycle. We think, therefore, that the concomitant stimulation of B- and T-cell responses is crucial for mounting an effective immunity against the dynamic and complex chain of events that accompany the HPV life cycle. Moreover, after virus clearance, such combined vaccines would shield epithelial cells from recurrent HPV infections. Appreciation of the scope and potential benefits of this kind of comprehensive anti-HPV weapon has driven the search for effective dual-purpose vaccines capable of a prophylactic and therapeutic action against HPV.

One approach in this direction is to use VLPs as carriers of early HPV antigens. VLPs containing an L2-E7 fusion protein, and thus capable of inducing anti-L1 prophylactic and anti-E7 therapeutic responses, have been produced and tested in mouse models of HPV-induced carcinogenesis [18, 38]. Another approach was to produce chimeric VLPs composed of the HPV16 L1 protein, C-terminally fused to the N-terminal region of HPV16 E7 [16]. After promising preclinical prophylactic and therapeutic effects [39, 40], this combined vaccine was administered to patients with HPV-induced CIN 2/3 lesions, but no significant clinical effects were observed [41]. Another combined antigen, TA-CIN (tissue antigen-cervical intraepithelial neoplasia) containing HPV16 L2, E6 and E7 was applied to vulvar intraepithelial neoplasia patients together with the innate immunity modulator Imiquimod [19]. The trial was not placebo-controlled, but lesions regressed more frequently compared to previous intervention studies and a local infiltration of CD4^+^ and CD8^+^ T cells was observed.

In the present work, we aimed to generate a therapeutic antigen with broadly protective ability by combining L2 from various HPV types and E7 from HPV16. Our concept of vaccine design followed an increasing complexity development pipeline. Initially, we validated the ability of our monomeric PADRE-supplemented Trx scaffold to induce cytotoxic T-cell responses against the OVA CTL epitope, utilized as a prototype target epitope, and subsequently confirmed also for the HPV16 E7 CTL epitope. Building on our previous work, we showed that multiple prophylactic epitopes as well as a three-fold repeated HPV16 E7 CTL epitope could be incorporated into the basic Trx scaffold, which upon fusion with the OVX313 hepatmerization domain was converted into a highly multivalent nanoparticle-like format. The resulting antigen, Trx-8mer-flank E7-OVX313, was then shown to be capable of inducing HPV neutralizing responses as well as potent antitumor effects in C57BL/6N mice.

In addition to the combined prophylactic and therapeutic activity, our dual-purpose vaccine presents some unique advantages compared to previous similar HPV vaccines. One of the main aims that inspired and motivated Trx-8mer-flank E7-OVX313 development was, in fact, the reduction of the economic burden on global health. Our vaccine was produced in *E*. *coli*, which is the most cost-effective and easy-to-use recombinant protein production host. Further, the Trx scaffold utilized for our antigen is derived from the hyperthermophile *Pyrococcus furiosus*, and it has previously been shown to be an extremely robust protein, highly resistant to heat, freeze-drying as well as proteolytic damage, and with a large capacity to accept long polypeptide inserts within its display site [30, 31, 42]. Due to this sturdy scaffold, our vaccine, a prophylactic variant of which is currently undergoing GMP production in view of a phase I clinical study, does not require cold-chain transportation, which cuts costs significantly and makes it applicable to many underdeveloped countries worldwide. In addition, it was reported that T-helper responses against E7 are considerably correlated with self-regression of HPV-induced lesions in healthy individuals [43, 44]. We thus envision a possible application of our vaccine in individuals with early-stage HPV infection who could possibly benefit from a boosting of E7-specific T-helper responses for the clearing of HPV-transformed cells.

We found that a 10-amino acids extension of the published CTL epitope [24], from RAHVYNIVTF (E7_49-57_) to QAEPDRAHVYNIVTFCCKCD (E7_44-62_, namely flank E7), significantly improved E7-specifc T-cell responses. We believe that this enhanced T-cell response can be attributed to the sequences flanking the CTL epitopes, which may facilitate proteasomal processing, thus promoting epitope presentation. Indeed, there have been several claims on the importance of the design of the linkers or flanking sequences interposed between individual epitopes for optimal vaccine immunogenicity and the induction of enhanced T-cell responses [45–49]. This is especially important if the vaccine harbors multi-epitopes. In addition to the inter-epitope flanking sequences, we have shown that the oligomerization domain OVX313 plays a pivotal role in overall immunogenicity. OVX313 is a positively charged derivative of the C4-binding protein (C4 bp) involved in complement system inhibition that promotes the autonomous heptamerization of any protein fusion partner. Also, it is a chimeric variant of avian C4 bp sharing less than 20% similarity with human C4 bp, a feature that has been engineered in order to minimize auto-antibody induction. It contains an amphipathic α-helix region, which is necessary and sufficient for heptamerization, as well as two cysteine residues which upon inter-subunit disulfide bond formation covalently stabilize the resulting heptameric structure [50]. OVX313 has been successfully employed for the construction of multiple prophylactic vaccines in addition to HPV [25–29, 51], some of which are progressing into clinical trial investigation studies (e.g., tuberculosis vaccine, NCT01879163; malaria vaccine, NCT02532049; influenza vaccine, NCT03594890). Due to the density of positive charges it provides, the OVX313 module may also enhance overall immunogenicity by promoting cellular internalization of the antigen. However, the immunological mechanisms underlying OVX313-mediated immunogenicity enhancement are not fully understood. We observed a T-cell response induced by stimulation with the ‘OVX313-I5’ peptide in IFN-γ ELISpot assay. However, this response could not be identified as either CD8^+^ or CD4^+^ T cell-specific by intracellular cytokine staining. Nevertheless, because of the fairly large size (20 amino acids) of the stimulating peptide, we assume it represents a T-helper response. Additionally, a T-cell response induced by the same OVX313 peptide was observed in BALB/c [23] but also in A2DR1 (S2 Fig) mice. This strongly suggests that the ‘OVX313-I5’ peptide is likely a universal T-helper epitope.

We observed enhanced OVA specific T-cell responses upon incorporation of the OVA-specific CTL epitope within the L2 8mer polytope displayed on Trx. Nonetheless, by employing a set of L2 8mer-specific, 20mer-long, overlapping peptides we could not identify any T-helper epitope clearly associated to the L2 polytope. Other assays will be required in order to precisely localize T-helper epitopes in the L2 8mer.

Epitope spreading, that is the development of immune responses against epitopes non-cross-reactive with the original inducing epitope, has been observed in patients upon therapeutic HPV vaccination [52, 53]. The potent therapeutic effect exerted by the Trx-8mer-flank E7-OVX313 antigen in TC-1 tumor-bearing mice was accompanied by an epitope-specific anti-E7 CTL response without any detectable response against E6, thus suggesting that epitope spreading was not involved in TC-1 tumor regression.

There are also a few shortcomings and potential limitations associated with our combined vaccine. For example, at variance with the extremely broad prophylactic capacity conferred by the L2 polytope [23], the presence of a monotypic E7 epitope may limit its therapeutic activity to one (HPV16) or only a few HPV types. Also, our dual-purpose vaccine was developed and tested in the genetic background of C57BL/6N mice. Since our ultimate goal is to translate the Trx-8mer-flank E7-OVX313 prototype antigen from animals to humans, more efforts will have to be made toward the development of an HPV vaccine that can induce both humoral and cellular immune responses in a human genetic background.

## Materials and methods

### Ethics statement

C57BL/6N mice and BALB/c mice at the DKFZ are kept in compliance with German and European statutes and all animal experiments were carried out with the approval of the responsible Animal Ethics Committee (Regional Council of Karlsruhe, Germany; 35–9185.81/G-20/17 and 35–9185.81/G-248/16).

### Protein expression and purification

Synthetic DNA encoding target proteins were inserted into the pET 24 plasmid for expression in *E*. *coli* BL21. “PADRE-Trx” derived proteins contain dual 6xHis-tag and nickel affinity chromatography was applied for purification of these antigens. “OVX313-Trx” recombinant proteins were purified by cation exchange chromatography (HiTrap SP FF column, GE Healthcare) based on an arginine-rich motif at the C-terminus of the OVX313 heptamerization domain (OligoDOM technology, OSIVAX). The concentration and purity of the proteins were analyzed by SDS-PAGE–Coomassie blue staining. For endotoxin removal, all proteins were detoxified twice by Triton X-114 separation before immunization.

### Mouse immunization

The 6 to 8 weeks-old female C57BL/6N mice were purchased from Envigo (Gannet, France; animal permit G20/17, Regierungspräsidium Karlsruhe, Germany) and the 6 to 8 weeks-old female BALB/c mice were obtained from Charles River Laboratories (Sulzfeld, Germany; animal permit G248/16, Regierungspräsidium Karlsruhe, Germany). Mice were kept at the German Cancer Research Center under specific-pathogen-free conditions. C57BL/6N mice were used for assessment of cellular immune responses. Twenty μg of the protein adjuvanted with 50% (vol/vol) AddaVax (InvivoGen; 50 μl) or Incomplete Freund’s adjuvant (Sigma-Aldrich; 50 μl), or 50 μg aluminum hydroxide (Brenntag) and 10 μg synthetic monophosphoryl lipid A (Avanti Lipids) (5 μl aluminum hydroxide, 10 μl monophosphoryl lipid A, in a total volume of 50 μl) was injected subcutaneously (s.c.) into the base of a mouse tail. For peptide administration, the adjuvant formulation and the immunization route were the same as protein vaccination, but the injected dose was different. A peptide mixture containing 100 μg MHC-I restricted peptide and 140 μg MHC-II restricted peptide was injected s.c. in a total volume of 50 μl. Mice received a single injection and were sacrificed 7 days later for *ex vivo* analysis of T-cell responses by IFN-γ ELISpot assay. Alternatively, mice were immunized twice at weekly intervals and splenocytes of sacrificed mice were analyzed by multimer staining 7 days later after the last immunization. Female BALB/c mice were employed for evaluation of humoral immune responses. Twenty μg of protein adjuvanted with 50% (vol/vol) AddaVax (25 μl) were injected intramuscularly (i.m.) into the caudal thigh muscle. Mice were immunized 4 times at biweekly intervals. One month after the last immunization, blood was collected by cardiac puncture and serum was analyzed against HPV16 and HPV18 pseudovirions using L1-PBNA.

### Pseudovirion preparation

Pseudovirions (PsV) were prepared by cotransfection of the human fibroblast cell line 293TT with plasmids encoding humanized HPV L1 and L2 proteins of different HPV types plus a reporter plasmid. Purification was performed by iodixanol gradient ultracentrifugation following a previously described protocol [54, 55].

### *In vitro* standard (L1) neutralization assay (L1-PBNA)

Unless otherwise stated, the L1-PBNA was used to detect neutralizing antibodies in sera against human papillomaviruses. L1-PBNA was performed as described previously [54]. Briefly, 50 μl of serially diluted sera were combined with 50 μl of diluted PsV and incubated at room temperature for 20 min. Next, 50 μl of HeLa T cells [56] (2.5×10^5^ cells/ml) were added to the PsV-antibody mixture and incubated for 48 h at 37°C humidified incubator. The amount of secreted Gaussia luciferase in 10 μl of cell culture supernatant was determined using the Gaussia glow juice kit according to the manufacturer's instructions (PJK GmbH; Germany). The light emissions of samples were measured 15 minutes after substrate addition. Serum concentrations (titers) inhibiting 50% of the PsV infection (EC50) were calculated from median of duplicates. EC50 values > 50 were defined as neutralizing positive.

### *In vivo* neutralization in the cervico-vaginal mouse model

The mouse cervico-vaginal model experiment was performed as described previously [57] with minor modifications (animal permit number G-93/17). The passive transfer assay was performed over the course of 8 days. On day 1, BALB/c male mouse cage bedding was transferred to the cages of female mice to induce hormonal synchronization (Whitten effect). On day 3, 100 μl of 30 mg/ml medroxyprogesterone acetate (Depo-Provera; Pharmacia) was injected subcutaneously. One hundred microliters of serum from immunized mice (diluted 1:1 with PBS) was delivered i.p. to each mouse on day 5. On day 6, mice were treated intravaginally with 50 μl of 4% Nonoxynol-9 (N9; Spectrum) in 4% carboxymethyl cellulose (Sigma). Four hours after N9 treatment, HPV pseudovirions encapsidating a firefly luciferase plasmid in 4% carboxymethyl cellulose (Sigma) were instilled intravaginally. Luminescence-based imaging using a Xenogen *in vivo* imaging system (IVIS) imager (Xenogen Corporation; PerkinElmer) was performed on day 8, and the efficiency of L2-directed immune responses was assessed after intravaginal instillation of 20 μl luciferin substrate (15 mg/ml; Promega). A region-of-interest (ROI) analysis was performed using Living Image 2.50.1 software (Xenogen; PerkinElmer). Background signal was obtained and subtracted by imaging each group of mice prior to instillation of luciferin.

### Antibody isotype ELISA

Serocluster 96-well "U" bottom plates (Costar, USA) were coated with 100 μl of 1 mg/ml streptavidin (Sigma-Aldrich, Germany) overnight at 37°C. On the next day, N-terminally biotinylated-HPV16 L2 peptide (GGSGKTCKQAGTCPPDIIPKVEGK) and biotinylated-HPV18 L2 peptide (GGSGKTCKQSGTCPPDVVPKVEGT) (GenScript,New Jersey) were added to the plates at a concentration of 2.5 μg/ml. All serum samples were diluted 1:50 in PBS containing 1.5% milk and 0.3% Tween 20 and then diluted in a three-fold serial dilution down the plate. Horse-radish-peroxidase (HRP)-conjugated goat-anti-mouse IgG1, IgG2a, IgG2b, IgG3, IgA and IgM (Southern Biotech, USA) were used to determine the isotype of anti-L2 antibodies in sera. The colorimetric reaction was quantified at 405 nm with Multiskan Go (Thermo Fisher Scientific, USA).

### Enzyme-linked immunosorbent spot assay (ELISpot)

Splenocytes isolated from vaccinated mice were plated at 1 million cells per well with 100 ng/ml of the synthetic peptides OVA_257-264_, E7_49-57,_ E6_48-57_, PADRE (GenScript, New Jersey) or 10 μg/ml of OVX313 peptide panel (20mer-peptide set with 12 amino acids overlap covering the entire OVX313 sequence: OVX313-I1, EVGRQNLIRSKEEILKKLKE; OVX313-I2, RSKEEILKKLKELQEGSKKQ; OVX313-I3, KLKELQEGSKKQGDADVCGE; OVX313-I4, SKKQGDADVCGEVAYIQSVV; OVX313-I5, VCGEVAYIQSVVSDCHVPTA; OVX313-I6, QSVVSDCHVPTAELRTLLEI, OVX313-I7, VPTAELRTLLEIRKLFLEIQ; OVX313-I8, LLEIRKLFLEIQKLKVEGRR; OVX313-I9, RKLFLEIQKLKVEGRRRRRS) (Mimotopes, Australia) or 5×10^4^ target cells (EG7, EL4, RMA, RMA/E7 or TC-1 cells) in anti-mouse IFN-γ (BD Pharmingen, USA)-coated Multiscreen IP plates (Merck Millipore, Germany). Concanavaline A (10 μg /ml) and un-stimulated splenocytes were used as positive and negative controls, respectively. Secreted IFN-γ was detected using biotinylated anti-mouse IFN-γ antibody (BD Pharmingen, USA), alkaline phosphatase (AKP)-streptavidin conjugate (BD Pharmingen, USA), and staining with 1-Step nitroblue tetrazolium (NBT)–5-bromo-4-chloro-3-indolylphosphate (BCIP) substrate (Sigma, USA). The number of IFN-γ specific spots was counted using an ELISpot reader (AID, Strassberg, Germany), calculated by subtracting the average negative control value, and generated as the net number of spot-forming units (SFUs).

### Tetramer and streptamer staining

APC-conjugated H2-K^b^/OVA_257-264_ tetramer (MBL, USA) or APC-conjugated H2-D^b^/E7_49-57_ streptamer (IBA, Germany) was incubated with 2 million splenocytes per well at dark for 45 minutes. Next, the cells were stained with PE-labeled CD8-α specific antibody (KT15, Santa Cruz Biotechnology, Germany) and Live/Dead yellow dye (Thermo Fisher Scientific, Germany) in FACS buffer at 4°C for 1 hour protected from the light. The number of OVA_257-264_ tetramer^+^ CD8-α^+^ or E7_49-57_ streptamer^+^ CD8-α^+^ T cells was determined by flow cytometry.

### Tumor regression assay

Six weeks-old C57BL/6N female mice were inoculated with 0.2×10^6^ TC-1 cells in 100 μl of PBS on their right flank. When tumor size was between 3–5 mm diameter, half of the tumor bearing animals were immunized at the base of the tail s.c. with 20 μg of antigen Trx-8mer-flank E7-OVX313 adjuvanted with 50% (vol/vol) AddaVax (50 μl), and two doses of the vaccine were received with 5 days apart. The rest of tumor bearing mice were used as negative control without any vaccination. When the tumor of vaccinated mice had completely regressed, half of the regressors were re-challenged with 0.3×10^6^ TC-1 cells to detect the memory T-cell response induced by the vaccine. Tumor volume was measured with a digital caliper. Mice were excluded from the experiment when the tumor diameter exceeded over 1.5 cm.

### Statistical analysis

Statistical significance was calculated with the nonparametric Mann-Whitney test performed with GraphPad Prism 8.0 (GraphPad Software, USA). P ≤ 0.05 was considered statistically significant.

## Supporting information

S1 Fig IFA is a superior adjuvant for peptide immunization. Numbers of IFN-γ spots per 10^6^ splenocytes are compared among groups of mice immunized with OVA_257-264_ and PADRE peptide mix either with IFA or AddaVax as adjuvant. Splenocytes were stimulated *in vitro* with the OVA_257-264_ peptide. Shown are the mean and SD of triplicate values on each mouse. P-value ≤ 0.05 was considered as significant and are labeled as follows: *, P-value < 0.05; **, P-value < 0.01; ***, P-value < 0.001; ****, P-value < 0.0001.
(TIF)

S2 Fig A T-helper response is induced by the ‘OVX313-I5’ peptide in A2DR1 mice. Mice were immunized twice at 5 days intervals with the Trx-8mer-flank E7_11-19_-OVX313 antigen. Splenocytes were stimulated with the OVX313-I5 peptide *in vitro*. Data, representing the numbers of IFN-γ spots per 10^6^ splenocytes, are the mean and SD of triplicate values on each mouse. E7_11-19_ is a HPV16 E7-derived CTL epitope (YMLDLQPET, HLA-A2^+^ restricted) [58]. Flank E7_11-19_ is the extended version of the E7_11-19_ epitope, flanked on both sides by the five amino acids that in the HPV16 E7 protein are located upstream (PTLHE) and downstream (TDLYC) to the sequence of the E7_11-19_ epitope (YMLDLQPET).
(TIF)

## References

[1] Ferlay J, Colombet M, Soerjomataram I, Mathers C, Parkin DM. Estimating the global cancer incidence and mortality in 2018: GLOBOCAN sources and methods. 2019;144(8):1941–53. 10.1002/ijc.31937 . 30350310

[2] Bray F, Ferlay J, Soerjomataram I, Siegel RL, Torre LA, Jemal A. Global cancer statistics 2018: GLOBOCAN estimates of incidence and mortality worldwide for 36 cancers in 185 countries. CA: a cancer journal for clinicians. 2018;68(6):394–424. Epub 2018/09/13. 10.3322/caac.21492 . 30207593

[3] IARC [Internet]. Global Cancer Observatory (GLOBOCAN) Cancer Tomorrow 2018 Estimates. https://gco.iarc.fr/tomorrow/home.

[4] zur Hausen H. Papillomaviruses in the causation of human cancers—a brief historical account. Virology. 2009;384(2):260–5. Epub 2009/01/13. 10.1016/j.virol.2008.11.046 . 19135222

[5] Munoz N, Bosch FX, Castellsague X, Diaz M, de Sanjose S, Hammouda D, et al Against which human papillomavirus types shall we vaccinate and screen? The international perspective. International journal of cancer. 2004;111(2):278–85. Epub 2004/06/16. 10.1002/ijc.20244 . 15197783

[6] zur Hausen H. Papillomaviruses and cancer: from basic studies to clinical application. Nature reviews Cancer. 2002;2(5):342–50. Epub 2002/06/05. 10.1038/nrc798 . 12044010

[7] Lehtinen M, Dillner J. Clinical trials of human papillomavirus vaccines and beyond. Nature reviews Clinical oncology. 2013;10(7):400–10. Epub 2013/06/06. 10.1038/nrclinonc.2013.84 . 23736648

[8] Huh WK, Joura EA, Giuliano AR, Iversen OE, de Andrade RP, Ault KA, et al Final efficacy, immunogenicity, and safety analyses of a nine-valent human papillomavirus vaccine in women aged 16–26 years: a randomised, double-blind trial. Lancet (London, England). 2017;390(10108):2143–59. Epub 2017/09/10. 10.1016/s0140-6736(17)31821-4 . 28886907

[9] Hildesheim A, Herrero R, Wacholder S, Rodriguez AC, Solomon D, Bratti MC, et al Effect of human papillomavirus 16/18 L1 viruslike particle vaccine among young women with preexisting infection: a randomized trial. Jama. 2007;298(7):743–53. Epub 2007/08/21. 10.1001/jama.298.7.743 . 17699008

[10] Garland SM, Hernandez-Avila M, Wheeler CM, Perez G, Harper DM, Leodolter S, et al Quadrivalent vaccine against human papillomavirus to prevent anogenital diseases. The New England journal of medicine. 2007;356(19):1928–43. Epub 2007/05/15. 10.1056/NEJMoa061760 . 17494926

[11] World Health Organization [Internet]. MI4A: GLOBAL MARKET STUDY-HPV. https://www.who.int/immunization/programmes_systems/procurement/mi4a/platform/module2/WHO_HPV_market_study_public_summary.pdf.

[12] Lin K, Doolan K, Hung CF, Wu TC. Perspectives for preventive and therapeutic HPV vaccines. Journal of the Formosan Medical Association = Taiwan yi zhi. 2010;109(1):4–24. Epub 2010/02/04. 10.1016/s0929-6646(10)60017-4 . 20123582 PMC2908016

[13] Cordeiro MN, De Lima RCP, Paolini F, Melo A, Campos APF, Venuti A. Current research into novel therapeutic vaccines against cervical cancer. 2018;18(4):365–76. 10.1080/14737140.2018.1445527 . 29475377

[14] Roden RB, Ling M, Wu TC. Vaccination to prevent and treat cervical cancer. Human pathology. 2004;35(8):971–82. Epub 2004/08/07. 10.1016/j.humpath.2004.04.007 . 15297964

[15] Schellenbacher C, Roden RBS, Kirnbauer R. Developments in L2-based human papillomavirus (HPV) vaccines. Virus research. 2017;231:166–75. Epub 2016/11/28. 10.1016/j.virusres.2016.11.020 . 27889616 PMC5549463

[16] Muller M, Zhou J, Reed TD, Rittmuller C, Burger A, Gabelsberger J, et al Chimeric papillomavirus-like particles. Virology. 1997;234(1):93–111. Epub 1997/07/21. 10.1006/viro.1997.8591 . 9234950

[17] Kirnbauer R, Taub J, Greenstone H, Roden R, Durst M, Gissmann L, et al Efficient self-assembly of human papillomavirus type 16 L1 and L1-L2 into virus-like particles. Journal of virology. 1993;67(12):6929–36. Epub 1993/12/01. . 8230414 10.1128/jvi.67.12.6929-6936.1993 PMC238150

[18] Greenstone HL, Nieland JD, de Visser KE, De Bruijn ML, Kirnbauer R, Roden RB, et al Chimeric papillomavirus virus-like particles elicit antitumor immunity against the E7 oncoprotein in an HPV16 tumor model. Proceedings of the National Academy of Sciences of the United States of America. 1998;95(4):1800–5. Epub 1998/03/21. 9465097 10.1073/pnas.95.4.1800 PMC19193

[19] van der Burg SH, Kwappenberg KM, O'Neill T, Brandt RM, Melief CJ, Hickling JK, et al Pre-clinical safety and efficacy of TA-CIN, a recombinant HPV16 L2E6E7 fusion protein vaccine, in homologous and heterologous prime-boost regimens. Vaccine. 2001;19(27):3652–60. Epub 2001/06/08. 10.1016/s0264-410x(01)00086-x . 11395199

[20] Kim D, Gambhira R, Karanam B, Monie A, Hung CF, Roden R, et al Generation and characterization of a preventive and therapeutic HPV DNA vaccine. Vaccine. 2008;26(3):351–60. Epub 2007/12/22. 10.1016/j.vaccine.2007.11.019 18096279 PMC2258233

[21] Xu YF, Wang QY, Zhang HT, Han YH, Song GX, Xu XM. Encapsidating artificial human papillomavirus-16 mE7 protein in human papillomavirus-6b L1/L2 virus like particles. Chinese medical journal. 2007;120(6):503–8. Epub 2007/04/19. . 17439745

[22] Spagnoli G, Pouyanfard S, Cavazzini D, Canali E, Maggi S, Tommasino M, et al Broadly neutralizing antiviral responses induced by a single-molecule HPV vaccine based on thermostable thioredoxin-L2 multiepitope nanoparticles. Scientific reports. 2017;7(1):18000 Epub 2017/12/23. 10.1038/s41598-017-18177-1 29269879 PMC5740060

[23] Pouyanfard S, Spagnoli G, Bulli L, Balz K, Yang F, Odenwald C, et al Minor Capsid Protein L2 Polytope Induces Broad Protection against Oncogenic and Mucosal Human Papillomaviruses. Journal of virology. 2018;92(4). Epub 2017/12/08. 10.1128/jvi.01930-17 29212932 PMC5790957

[24] Feltkamp MC, Smits HL, Vierboom MP, Minnaar RP, de Jongh BM, Drijfhout JW, et al Vaccination with cytotoxic T lymphocyte epitope-containing peptide protects against a tumor induced by human papillomavirus type 16-transformed cells. European journal of immunology. 1993;23(9):2242–9. Epub 1993/09/01. 10.1002/eji.1830230929 . 7690326

[25] Del Campo J, Pizzorno A. OVX836 a recombinant nucleoprotein vaccine inducing cellular responses and protective efficacy against multiple influenza A subtypes. 2019;4:4 10.1038/s41541-019-0098-4 . 30701093 PMC6344521

[26] Ogun SA, Dumon-Seignovert L, Marchand JB, Holder AA, Hill F. The oligomerization domain of C4-binding protein (C4bp) acts as an adjuvant, and the fusion protein comprised of the 19-kilodalton merozoite surface protein 1 fused with the murine C4bp domain protects mice against malaria. Infection and immunity. 2008;76(8):3817–23. Epub 2008/05/14. 10.1128/iai.01369-07 18474650 PMC2493234

[27] Minhinnick A, Satti I, Harris S, Wilkie M, Sheehan S, Stockdale L, et al A first-in-human phase 1 trial to evaluate the safety and immunogenicity of the candidate tuberculosis vaccine MVA85A-IMX313, administered to BCG-vaccinated adults. Vaccine. 2016;34(11):1412–21. Epub 2016/02/09. 10.1016/j.vaccine.2016.01.062 26854906 PMC4786162

[28] Li Y, Leneghan DB, Miura K, Nikolaeva D, Brian IJ, Dicks MD, et al Enhancing immunogenicity and transmission-blocking activity of malaria vaccines by fusing Pfs25 to IMX313 multimerization technology. Scientific reports. 2016;6:18848 Epub 2016/01/09. 10.1038/srep18848 26743316 PMC4705524

[29] Spencer AJ, Hill F, Honeycutt JD, Cottingham MG, Bregu M, Rollier CS, et al Fusion of the Mycobacterium tuberculosis antigen 85A to an oligomerization domain enhances its immunogenicity in both mice and non-human primates. PloS one. 2012;7(3):e33555 Epub 2012/04/04. 10.1371/journal.pone.0033555 22470455 PMC3314664

[30] Seitz H, Canali E, Ribeiro-Muller L, Palfi A, Bolchi A, Tommasino M, et al A three component mix of thioredoxin-L2 antigens elicits broadly neutralizing responses against oncogenic human papillomaviruses. Vaccine. 2014;32(22):2610–7. Epub 2014/03/26. 10.1016/j.vaccine.2014.03.033 . 24662712

[31] Seitz H, Ribeiro-Muller L, Canali E, Bolchi A, Tommasino M, Ottonello S, et al Robust In Vitro and In Vivo Neutralization against Multiple High-Risk HPV Types Induced by a Thermostable Thioredoxin-L2 Vaccine. Cancer prevention research (Philadelphia, Pa). 2015;8(10):932–41. Epub 2015/07/15. 10.1158/1940-6207.capr-15-0164 . 26170394

[32] Alexander J, Sidney J, Southwood S, Ruppert J, Oseroff C, Maewal A, et al Development of high potency universal DR-restricted helper epitopes by modification of high affinity DR-blocking peptides. Immunity. 1994;1(9):751–61. Epub 1994/12/01. . 7895164 10.1016/s1074-7613(94)80017-0

[33] Rubio I, Bolchi A, Moretto N, Canali E, Gissmann L, Tommasino M, et al Potent anti-HPV immune responses induced by tandem repeats of the HPV16 L2 (20–38) peptide displayed on bacterial thioredoxin. Vaccine. 2009;27(13):1949–56. Epub 2009/04/17. 10.1016/j.vaccine.2009.01.102 . 19368776

[34] Lin KY, Guarnieri FG, Staveley-O'Carroll KF, Levitsky HI, August JT, Pardoll DM, et al Treatment of established tumors with a novel vaccine that enhances major histocompatibility class II presentation of tumor antigen. Cancer research. 1996;56(1):21–6. Epub 1996/01/01. . 8548765

[35] Ribas A, Timmerman JM, Butterfield LH, Economou JS. Determinant spreading and tumor responses after peptide-based cancer immunotherapy. Trends in immunology. 2003;24(2):58–61. Epub 2003/01/28. 10.1016/s1471-4906(02)00029-7 . 12547500

[36] Shibata T, Lieblong BJ, Sasagawa T, Nakagawa M. The promise of combining cancer vaccine and checkpoint blockade for treating HPV-related cancer. Cancer treatment reviews. 2019;78:8–16. Epub 2019/07/16. 10.1016/j.ctrv.2019.07.001 31302573 PMC6710123

[37] Oosterhuis K, Ohlschlager P, van den Berg JH, Toebes M, Gomez R, Schumacher TN, et al Preclinical development of highly effective and safe DNA vaccines directed against HPV 16 E6 and E7. International journal of cancer. 2011;129(2):397–406. Epub 2011/01/06. 10.1002/ijc.25894 . 21207427

[38] De Bruijn ML, Greenstone HL, Vermeulen H, Melief CJ, Lowy DR, Schiller JT, et al L1-specific protection from tumor challenge elicited by HPV16 virus-like particles. Virology. 1998;250(2):371–6. Epub 1998/10/30. 10.1006/viro.1998.9372 . 9792847

[39] Schäfer K, Müller M, Faath S, Henn A, Osen W, Zentgraf H, et al Immune response to human papillomavirus 16 L1E7 chimeric virus-like particles: Induction of cytotoxic T cells and specific tumor protection. International journal of cancer. 1999;81(6):881–8. 10.1002/(sici)1097-0215(19990611)81:6<881::aid-ijc8>3.0.co;2-t . 10362134

[40] Öhlschläger P, Osen W, Dell K, Faath S, Garcea RL, Jochmus I, et al, Human Papillomavirus Type 16 L1 Capsomeres Induce L1-Specific Cytotoxic T Lymphocytes and Tumor Regression inC57BL/6 Mice. Journal of Virology. 2003;77(8):4635–45. 10.1128/jvi.77.8.4635-4645.2003. . 12663770 PMC152157

[41] Kaufmann AM, Nieland JD, Jochmus I, Baur S, Friese K, Gabelsberger J, et al Vaccination trial with HPV16 L1E7 chimeric virus-like particles in women suffering from high grade cervical intraepithelial neoplasia (CIN 2/3). International journal of cancer. 2007;121(12):2794–800. Epub 2007/08/28. 10.1002/ijc.23022 . 17721997

[42] Canali E, Bolchi A, Spagnoli G, Seitz H, Rubio I, Pertinhez TA, et al A high-performance thioredoxin-based scaffold for peptide immunogen construction: proof-of-concept testing with a human papillomavirus epitope. Scientific reports. 2014;4:4729 Epub 2014/04/23. 10.1038/srep04729 24751665 PMC3994442

[43] Kadish AS, Timmins P, Wang Y, Ho GY, Burk RD, Ketz J, et al Regression of cervical intraepithelial neoplasia and loss of human papillomavirus (HPV) infection is associated with cell-mediated immune responses to an HPV type 16 E7 peptide. Cancer epidemiology, biomarkers & prevention: a publication of the American Association for Cancer Research, cosponsored by the American Society of Preventive Oncology. 2002;11(5):483–8. Epub 2002/05/16. . 12010863

[44] Koskimaa HM, Paaso A, Welters MJP, Grenman S, Syrjanen K, van der Burg SH, et al The presence of human papillomavirus (HPV) in placenta and/or cord blood might result in Th2 polarization. European journal of clinical microbiology & infectious diseases: official publication of the European Society of Clinical Microbiology. 2017;36(8):1491–503. Epub 2017/03/23. 10.1007/s10096-017-2958-z 28324192 PMC5524867

[45] de Oliveira LM, Morale MG, Chaves AA, Cavalher AM, Lopes AS, de Diniz O, et al Design, Immune Responses and Anti-Tumor Potential of an HPV16 E6E7 Multi-Epitope Vaccine. PloS one. 2015;10(9):e0138686 Epub 2015/09/22. 10.1371/journal.pone.0138686 26390407 PMC4577214

[46] Velders MP, Weijzen S, Eiben GL, Elmishad AG, Kloetzel PM, Higgins T, et al Defined flanking spacers and enhanced proteolysis is essential for eradication of established tumors by an epitope string DNA vaccine. Journal of immunology (Baltimore, Md: 1950). 2001;166(9):5366–73. Epub 2001/04/21. . 11313372 10.4049/jimmunol.166.9.5366

[47] Levy A, Pitcovski J, Frankenburg S, Elias O, Altuvia Y, Margalit H, et al A melanoma multiepitope polypeptide induces specific CD8+ T-cell response. Cellular immunology. 2007;250(1–2):24–30. Epub 2008/02/16. 10.1016/j.cellimm.2008.01.001 18275944 PMC2413004

[48] Bartkowiak T, Singh S, Yang G, Galvan G, Haria D, Ai M, et al Unique potential of 4-1BB agonist antibody to promote durable regression of HPV+ tumors when combined with an E6/E7 peptide vaccine. Proceedings of the National Academy of Sciences of the United States of America. 2015;112(38):E5290–9. Epub 2015/09/10. 10.1073/pnas.1514418112 26351680 PMC4586868

[49] Yamada A, Sasada T, Noguchi M, Itoh K. Next-generation peptide vaccines for advanced cancer. Cancer science. 2013;104(1):15–21. Epub 2012/10/31. 10.1111/cas.12050 . 23107418 PMC7657262

[50] Kask L, Hillarp A, Ramesh B, Dahlback B, Blom AM. Structural requirements for the intracellular subunit polymerization of the complement inhibitor C4b-binding protein. Biochemistry. 2002;41(30):9349–57. Epub 2002/07/24. . 12135356 10.1021/bi025980+

[51] Tomusange K, Wijesundara D, Gummow J, Garrod T, Li Y, Gray L, et al A HIV-Tat/C4-binding protein chimera encoded by a DNA vaccine is highly immunogenic and contains acute EcoHIV infection in mice. Scientific reports. 2016;6:29131 Epub 2016/07/01. 10.1038/srep29131 27358023 PMC4928126

[52] Coleman HN, Greenfield WW, Stratton SL, Vaughn R, Kieber A, Moerman-Herzog AM, et al Human papillomavirus type 16 viral load is decreased following a therapeutic vaccination. Cancer immunology, immunotherapy: CII. 2016;65(5):563–73. Epub 2016/03/17. 10.1007/s00262-016-1821-x 26980480 PMC4841729

[53] Greenfield WW, Stratton SL, Myrick RS, Vaughn R, Donnalley LM, Coleman HN, et al A phase I dose-escalation clinical trial of a peptide-based human papillomavirus therapeutic vaccine with Candida skin test reagent as a novel vaccine adjuvant for treating women with biopsy-proven cervical intraepithelial neoplasia 2/3. Oncoimmunology. 2015;4(10):e1031439 Epub 2015/10/10. 10.1080/2162402x.2015.1031439 26451301 PMC4590015

[54] Seitz H, Dantheny T, Burkart F, Ottonello S, Muller M. Influence of oxidation and multimerization on the immunogenicity of a thioredoxin-l2 prophylactic papillomavirus vaccine. Clinical and vaccine immunology: CVI. 2013;20(7):1061–9. 10.1128/CVI.00195-13 . 23677323 PMC3697451

[55] Gambhira R, Karanam B, Jagu S, Roberts JN, Buck CB, Bossis I, et al A protective and broadly cross-neutralizing epitope of human papillomavirus L2. Journal of virology. 2007;81(24):13927–31. Epub 2007/10/12. 10.1128/jvi.00936-07 17928339 PMC2168823

[56] Sehr P, Rubio I, Seitz H, Putzker K, Ribeiro-Muller L, Pawlita M, et al High-throughput pseudovirion-based neutralization assay for analysis of natural and vaccine-induced antibodies against human papillomaviruses. PloS one. 2013;8(10):e75677 Epub 2013/10/15. 10.1371/journal.pone.0075677 24124504 PMC3790823

[57] Roberts JN, Buck CB, Thompson CD, Kines R, Bernardo M, Choyke PL, et al Genital transmission of HPV in a mouse model is potentiated by nonoxynol-9 and inhibited by carrageenan. Nature medicine. 2007;13(7):857–61. Epub 2007/07/03. 10.1038/nm1598 . 17603495

[58] Riemer AB, Keskin DB, Zhang G, Handley M, Anderson KS, Brusic V, et al A conserved E7-derived cytotoxic T lymphocyte epitope expressed on human papillomavirus 16-transformed HLA-A2+ epithelial cancers. The Journal of biological chemistry. 2010;285(38):29608–22. Epub 2010/07/10. 10.1074/jbc.M110.126722 . 20615877 PMC2937992

