# Supplementary figures and images for: Combined prophylactic and therapeutic immune responses against human papillomaviruses induced by a thioredoxin-based L2-E7 nanoparticle vaccine
Source: PLoS Pathog. 2020 Sep 4;16(9):e1008827. doi: 10.1371/journal.ppat.1008827 (PMC7498061; doi:10.1371/journal.ppat.1008827)

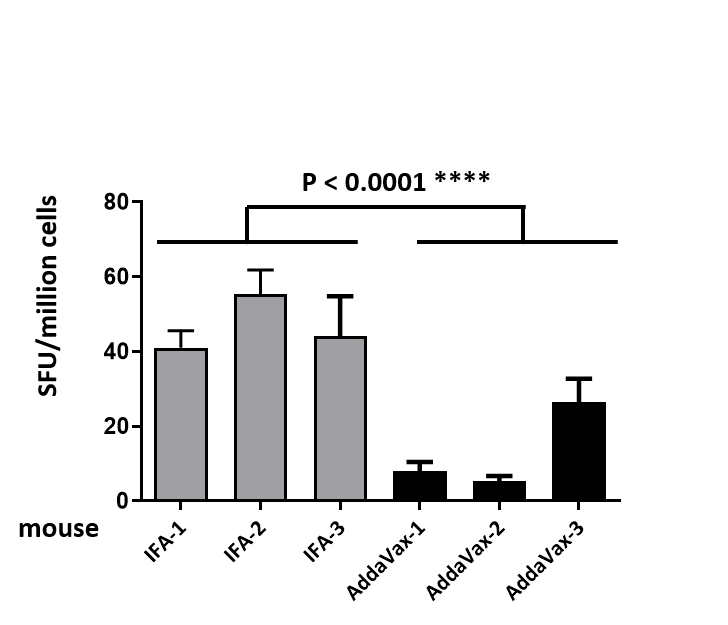

Supplement: S1 Fig — Numbers of IFN-γ spots per 106 splenocytes are compared among groups of mice immunized with OVA257-264 and PADRE peptide mix either with IFA or AddaVax as adjuvant. Splenocytes were stimulated in vitro with the OVA257-264 peptide. Shown are the mean and SD of triplicate values on each mouse. P-value ≤ 0.05 was considered as significant and are labeled as follows: *, P-value < 0.05; **, P-value < 0.01; ***, P-value < 0.001; ****, P-value < 0.0001. (TIF) [file ppat.1008827.s001.tif]

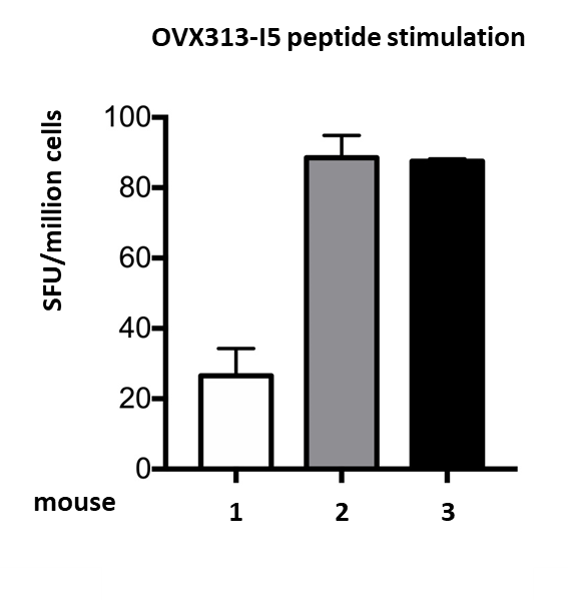

Supplement: S2 Fig — Mice were immunized twice at 5 days intervals with the Trx-8mer-flank E711-19-OVX313 antigen. Splenocytes were stimulated with the OVX313-I5 peptide in vitro. Data, representing the numbers of IFN-γ spots per 106 splenocytes, are the mean and SD of triplicate values on each mouse. E711-19 is a HPV16 E7-derived CTL epitope (YMLDLQPET, HLA-A2+ restricted) [58]. Flank E711-19 is the extended version of the E711-19 epitope, flanked on both sides by the five amino acids that in the HPV16 E7 protein are located upstream (PTLHE) and downstream (TDLYC) to the sequence of the E711-19 epitope (YMLDLQPET). (TIF) [file ppat.1008827.s002.tif]
